# Bo-Net: Deep learning-based model for automatic bone stromal cell segmentation of fluorescence microscopy images

**DOI:** 10.1371/journal.pone.0353796

**Published:** 2026-08-04

**Authors:** Giorgio Allegri, Luca Pavirani, Sergio Barrios, Sofia Rosy Caterina Sorice, Giulia Alessandrelli, Luca Marsilio, Antonios Mikos, Pietro Cerveri, Stefano Casarin, Eleonora Dondossola

**Affiliations:** 1 Department of Electronics, Information and Bioengineering, Polytechnic University of Milan, Milan, Italy; 2 Center for Precision Surgery, Houston Methodist Research Institute, Houston, Texas, United States of America; 3 David H. Koch Center for Applied Research of Genitourinary Cancers and Genitourinary Medical Oncology Department, The University of Texas MD Anderson Cancer Center, Houston, Texas, United States of America; 4 Rice University, Dept. of Bioengineering, Houston, Texas, United States of America; 5 Department of Surgery, Houston Methodist Hospital, Houston, Texas, United States of America; 6 LaSIE, UMR CNRS, La Rochelle Université, La Rochelle, France; Versiti Blood Research Institute, UNITED STATES OF AMERICA

## Abstract

**Background and objectives:**

Bone is a multicellular organ that is the site of complex pathophysiological events (such as cancer). 3D confocal and multiphoton microscopy, followed by manual analysis and quantification, allow the identification of molecular, cellular, and tissue mechanisms in their original context, enabling the investigation of spatial biology at subcellular level. The automation of these analyses is becoming increasingly important due to their time-consuming nature, lack of standardization, and inter-subject variability.

**Methods:**

Deep learning applied to image analysis could overcome current limitations and optimize preclinical research. This includes automatic semantic segmentation of bone cells (osteoblasts, osteoclasts, and blood vessels) and mineral component, followed by parameter extraction and quantification. Accordingly, fluorescence microscopy images were generated, pre-processed, and fed (total 21,395 images: 17,104 for training, 4291 for validation) into a neural network-based architecture named Bo-Net.

**Results:**

The fully automatic Bo-Net achieved strong performance across the tested metrics and segmentation accuracy similar to experienced biologists (R = 0.81–0.99 for parameters tested) and improved analysis time from days to seconds in a variety of experimental conditions, confirming its relevance in different biologically relevant contexts.

**Conclusions:**

As a result, a fully automated, yet reliable, tool to optimize the analysis of multiparametric fluorescence microscopy images available to the bone research community was generated.

## Introduction

Bone is a complex organ that provides mechanical support and protection to soft organs, maintains mineral homeostasis, and is the major site of hematopoiesis [1]. Its multicellular milieu includes immune cells at different stages of maturation, nerves, adipocytes, endothelial cells, bone-forming (osteoblasts, OBs), and bone-resorbing cells (osteoclasts, OCs) [1,2]. OBs, OCs, and endothelial cells play a relevant pathophysiological role in bone diseases, including primary tumors and metastasis, a lethal complication for patients with a variety of tumors, including prostate, renal, breast, and lung tumors, and melanoma [3–6]. OBs and OCs maintain bone homeostasis through a tightly balanced process of bone deposition and resorption [1,2,7]; this equilibrium is disrupted by tumor cells, which induce uncontrolled OB or OC activity, leading to osteoblastic or osteolytic lesions, respectively [6,8]. Besides fostering tumor growth and therapeutic resistance, this process results in pain, hypercalcemia, spinal cord compression, fractures, and limited mobility [4,9]. Endothelial cells, which form sinusoids and micro-vessels, supply oxygen and nutrients and allow hematopoietic cell trafficking. However, they also favor extravasation of cancer cells and support tumor cell growth through neoangiogenesis [3]. Therefore, studying OB, OC and blood vessel biology in bone is critical to understand tumor progression and its response to therapy.

Preclinical mouse models combined with three-dimensional (3D) multiphoton or confocal microscopy analysis, in vivo or ex vivo, provide spatial and temporal resolved information on bone biology, bone tumor progression, and therapy response, at the single-cell level and with subcellular resolution [10–13]. Using this approach, we monitored osteolytic progression, the kinetics of osteoclasts and bone resorption, and the effects of bisphosphonate therapy in metastatic prostate and breast cancer in bone [14]. We further detailed the therapeutic response (and resistance) to radium 223 radioisotope, providing both mechanistic insights into its mode of action and new strategies to improve its efficacy [15–17]. As a result, these preclinical strategies proved relevant to address the mechanisms of response and resistance of metastatic cells to bone, including the impact of cancer on bone health [14]. Microscopy acquisition, however, generates a large amount of complex, high-content biological images that must be reconstructed and elaborated, followed by data mining and quantification, a process that is often overall inefficient [18]. Due to the different properties of the bone cellular and extracellular components (including dimension, shape, and geometry complexity), no automatic tool is readily available to detect and quantify these data. Therefore, most of these analyses have been done manually by highly trained personnel, including individual cell counting by eye inspection, manual measurement of distances, and qualitative identification of cellular subtypes, or only partially automated, implying several disadvantages. As a result, the extraction of crucial information from these images is affected by human error, inter- and intra-observer variability, lack of standardization, and it is extremely time-consuming [19]. To both reduce the time required for analysis and to minimize human error, the need for automated analysis is urgent.

In this context, application of computer methods based on artificial intelligence (AI) to elaborate bioimages has gained significant importance, as they guarantee efficiency and objectiveness [20]. End-to-end deep learning is a recent AI-based paradigm for bioimage elaboration that reduces or even eliminates the need for hand-crafted feature detection and post-processing [21,22]. Images undergo direct processing through a multi-task, multi-layer encoder-decoder neural network, featuring one output path for each specific task. As a result, not only intermediate data (e.g., raw region segmentation), but also high-level annotated information (e.g., semantic patterns and quantification of geometrical/physical properties) are produced in the output. Deep learning algorithms improved image processing accuracy and reduced the time required to analyze microscopy images by automatizing the process of identification, recognition, extraction, and quantification of patterns and parameters of interest [20,23]. These models have great flexibility, and are employed in multiple applications such as cell and tissue segmentation, medical image analysis, and lesion detection [24,25]. In this domain, we have recently pioneered a tailored variant of the deep neural network U-Net specifically designed to process 3D multiphoton microscopy scans of a tumor-free, desmoplastic process called foreign body response [18]. This model automatically differentiated different structures of interest (fibroblast, immune cells, extracellular matrix, scaffold) with high accuracy [18].

Capitalizing on such prior achievements, in the present work we hypothesized that a trained computational algorithm can automatically segment and quantify microscopy images, significantly improving the study of bone biology. Accordingly, we proposed a deep learning-based infrastructure to optimize the time and improve the accuracy of image analysis of bone samples captured by fluorescence microscopy. The proposed end-to-end computational pipeline involved image data-cleaning and pre-processing, manual generation of ground-truth labels, model training and evaluation, and post-processing analyses to identify and quantify structures of interest (bone, OBs, OCs, blood vessels).

## Materials and methods

### Animal studies

Animal studies were approved by the Institutional Animal Care and Use Committee of the University of Texas, MD Anderson Cancer Center, which is accredited by the Association for Assessment and Accreditation of Laboratory Animal Care. All the studies were performed by trained personnel. Wild type mice were purchased by Jackson Lab; TRAP-tD-tomato mice were kindly provided by Dr. Masaru Ishii (Osaka University, Osaka, Japan); mCherry-Sp7 mice were kindly provided by Dr. Ralph Adams (Max Planck Institute for Molecular Biomedicine, Münster, Germany). Female or male mice, ≥ 8 weeks old, were housed with a maximum of five animals per cage in a state-of-the-art, air-conditioned, and specific pathogen–free animal facility and all procedures were performed in accordance with the NIH Policy on Humane Care and Use of Laboratory Animals. Tumor cell implantation was performed with mice under general anesthesia (isoflurane), and analgesia was provided at the end of each procedure (buprenorphine, 0.1 mg/kg). Tumor-bearing animals were observed daily and examined by a veterinarian 5 days/week for signs of morbidity (e.g., matted fur, weight loss, limited ambulation, and respiratory difficulty). In case of discomfort, the animals were euthanized within 24 hours by asphyxiation with carbon dioxide gas followed by cervical dislocation, consistent with the recommendations of the Panel on Euthanasia of the American Veterinary Medical Association. Tumor cells were administered in the tibia as reported [15,17]. This includes PC3 prostate cancer cells (ATCC), 318−1 osteosarcoma cells (established in the lab of Dr. G. Lozano, UT MD Anderson Cancer Center, Houston [26]) and RENCA renal cancer cells (ATCC) VHL [27] knockout. All the cells, tested regularly, were mycoplasma free. For osteoclast depletion experiment, clodronate treated and control groups (n = 5 mice/group) received intravenous injections of clodronate liposomes (5 mg/ml stock, 200 μl per mouse, Liposoma) or PSB control liposomes every 2 days for up to 2 weeks, as previously reported [28,29]. All animal studies have been conducted in compliance with ARRIVE guidelines.

### Sample staining and acquisition

Tumor free and tumor-bearing tibiae were collected, cleaned of soft tissue and muscle, and fixed for 24 hours in 4% paraformaldehyde in PBS. Bones were decalcified in 0.5M ethylenediamine tetraacetic acid (EDTA) solution for 6–7 days. Decalcified samples were embedded in 4% agarose and sectioned at 300-µm thick slices using a vibratome (Leica). This allows generating unique and biologically different slices, even if cut consecutively (given that the size of the murine cells of interest varies between 20–50 µm for osteoblasts, to 100 µm for osteoclasts, to 50–100 µm diameter for blood vessels [30]). Samples were blocked overnight in “staining solution” composed of 10% dimethyl sulfoxide (DMSO) (Sigma), 0.5% IGEPAL (Sigma), and 10% normal donkey serum (Southern Biotech) in PBS (further applied in the following steps). Samples were then incubated overnight at 4C with primary antibodies diluted 1:200 in staining solution. Primary antibodies included rat anti-endomucin (sc-65495, Santa Cruz), rabbit anti-laminin 1 + 2 (ab7463, Abcam), goat anti-mouse alkaline phosphatase (AF2910, R&D), and rabbit anti-tartrate resistant acid phosphatase (ab185716, Abcam). Samples were washed 5 times with excess PBS and incubated overnight with secondary antibodies (1:400) and DAPI (1:2000) in staining solution. Secondary antibodies were all purchased from Invitrogen and included donkey anti-rat AlexaFluor 488, donkey anti-rabbit AlexaFluor 680, donkey anti-goat AlexaFluor 594 or donkey anti-goat AlexaFluor 680. Samples were then washed 5 times with excess PBS prior to imaging.

Samples were acquired using a confocal microscope (Leica SP8) or a custom-made multiphoton microscope (LaVision BioTech).

The multiphoton microscope is equipped with three Ti:Sapphire lasers (Chameleon-XR, Coherent) and two optical parametric oscillators (APE/Coherent), resulting in a tunable excitation range (800–1300 nm). Images were acquired using a 16x water objective (numerical aperture, 0.8, Olympus) and multispectral detection was performed using up to five backward or two forward photomultipliers and up to three excitation wavelengths in two consecutive scans, to separate the following excitation and emission channels: AlexaFluor 488 (920 nm; 525/50 nm), AlexaFluor 594 (1090 nm; 595/40 nm), SHG (1090 nm; 525/50 nm), AlexaFluor 680 (1280 nm; 710/75 nm). Volumes of 360 × 360 μm (1064 × 1064 pixels; frequency 400; line average, 2; laser power 1–20%) with in-between slices z-depth of 8 μm, for a maximum depth of ~80–100 μm were acquired.

The Leica SP8 confocal microscope equipped with a Z6 APO zoom system. Images were acquired using a 20x objective at a resolution of 608 x 608 µm (1024 × 1024 pixels), with in-between slices z-depth of 8 μm, for a maximum depth of ~80–100 μm. Scanning was performed at a speed of 400 Hz, line average 1, pinhole set to 1 Airy Unit, and identical acquisition settings were maintained across all samples. Laser power (1–20%) was optimized for each fluorophore to achieve sufficient signal while avoiding saturation and photobleaching, and detector gain was adjusted accordingly.

The dataset was generated from a historical collection of multiphoton and confocal microscopy samples acquired over several years, including tissues derived from multiple fluorescent reporter mouse models and antibody-based stainings. This strategy enabled the inclusion of diverse imaging conditions and labelling approaches while minimizing the need for additional animals for model training. Following image-quality control and preprocessing, the final dataset used for model development comprised 90 multiphoton microscopy stacks and 85 confocal microscopy stacks. Each multiphoton microscopy stack included seven z-planes acquired across five channels, yielding 3,150 original single-channel images. Each confocal microscopy stack included seven z-planes acquired across four channels, yielding 2,380 original single-channel images. Seven consecutive intermediate z-planes (8 µm spacing; ~ 50 µm total depth) were selected for analysis to avoid superficial sectioning artifacts and signal loss in deeper regions, providing an optimal balance between tissue coverage and image quality for maximum-intensity projection within the same volume. Overall, the dataset comprised 5,530 original single-channel images before patch extraction and data augmentation. For OB and OC segmentation, maximum-intensity projections along the z-axis were used rather than individual z-slices to better capture the structures of interest. Overall, the dataset incorporated multiple biological conditions and imaging variations, which helped improve the robustness and generalizability of the model.

### Bo-Net Development

Bo-Net development included four consecutive phases: 1) initial image preprocessing, including cleaning and intensity transformation of the raw image data; 2) data preparation, including animal-level train-validation splitting, patch extraction, filtering, and data augmentation performed independently within each subset; 3) model training; and 4) post-processing to refine output predictions and extract quantitative readouts. The complete dataset comprised of 5,530 images (90 MPM stacks, 7 images/stack, 5 channels/image; 85 CM stacks, 7 images/stack, 4 channels/image). To better identify structures of interest for OB and OC we used maximum projection of the volumes over the z-axis rather than single images composing the stack. A schematic overview of the complete Bo-Net computational workflow is shown in S1 Fig.

1. Data Pre-processing
i. Pixel Normalization. Pixel values in all images were normalized to a [0,1] range. This process helped achieving a more uniform distribution across the dataset, minimizing the potential disparities in pixel value scale between different images, and mitigating risks such as exploding gradients. This normalization ensured consistent weight updates, enhancing algorithm stability and training efficiency (S2 Fig).
ii. Gamma Transformation. Image brightness was adjusted by using a power law transformation, (Equation 1).

$$\begin{array}{c} {\text{V}}_{\text{out}}=\;{\text{V}}_{\text{in}}^{\gamma } \end{array}$$ (1)

This processing enhances visibility of crucial features, enabling the algorithm to effectively distinguish between various structures. The selected gamma values for each structure, heuristically determined, are reported in Table 1. Although a limited number of saturated pixels were present in some images, segmentation was largely unaffected because it relies on morphological and spatial features rather than absolute intensity values. Preprocessing, normalization, and training with heterogeneous image intensities further enhanced robustness to variations in signal intensity and image quality.

**Table 1:** Chosen Gamma and Threshold values for each structure.

Channel	Gamma	Threshold
OB	1	100
OC	0.75	150
Bone	2	250
Vessels	1.5	200

iii. Histogram Equalization (stretching). A custom algorithm was implemented to redistribute the intensity values across the entire available range (S3 Fig).
iv. Black Image Removal. To exclude images that are not informative, which can lead to inaccurate predictions and suboptimal results, images with <2.5% of white pixels were removed from the pool.
v. Sample-wise pixel weighting. This pixel weighting procedure was specifically applied to the OC dataset, where accurate cell detection was complex due to the highly irregular shape. Each pixel was assigned a specific weight to optimize the separation of adjacent or closely related cells, as previously reported [18]. For each mask, a corresponding map was generated, associating a distinct value to each pixel.

Pixels belonging to OCs were assigned a weight of 0, indicating their significance in the classification process. Conversely, background pixels were weighted based on their proximity to the two nearest cells. The weight assigned to a background pixel was inversely proportional to its distance from these cells. As a result, pixels near cell boundaries were given higher weights, effectively emphasizing their importance during their training phase.

The weight (w_i,j_) assigned to each pixel x_i,j_, (pixel x located at position (i,j)), was determined using a Gaussian function applied to its Euclidean distance (D_i,j_) from the nearest cells. This Gaussian function provided a smooth and controlled decrease in weights with increasing distance from the cells, ensuring that the weights’ magnitudes were well-regulated. In formula:


$$\begin{array}{c} w_{i,j}=\left\{ \begin{array}{c} @l\text{0}\;if\;x_{i,j}\notin background\\ ae^{-\frac{D_{i,j}}{\text{2}\sigma^{\text{2}}}}\;otherwise \end{array}\right. \end{array}$$ (2)

with


$$\begin{array}{c} D_{i,j}={\left(d_{\text{1}}\left(x_{i,j}\right)+d_{\text{2}}\left(x_{i,j}\right)\right)}^{\text{2}} \end{array}$$ (3)

w_i,j_ is the weight of the pixel positioned in (i,j), *a* is the amplitude of the gaussian, empirically set to 100 and σ, the standard deviation, was set to 5 pixels, and finally, $d_{\text{1}}\left(x_{i,j}\right)$ and $d_{\text{2}}\left(x_{i,j}\right)$ are the distances of the pixel positioned in (i,j) from the 2 closest cells.

Through the integration of the pixel weighting strategy, we achieved remarkable enhancements in the algorithm’s capability to distinguish intricated cell structures within the OC dataset, leading to substantial improvements in classification accuracy. As a result, the algorithm’s overall performance was significantly bolstered, demonstrating superior efficacy across diverse datasets and challenging cell detection scenarios. In S4 Fig, the outcomes of the pixel weighting process are visually presented. The color mapping illustrates the significance assigned to each pixel: pixels with higher distances are denoted in blue, indicating their relatively lower importance, while pixels with the shortest distances are depicted in red, representing their heightened significance.

*Bone mask for CM images.* To obtain a bone mask after CM acquisition, that lacks detection of SHG, the OB, OC, blood vessel, and nucleus channel were superimposed. Areas where their signal was absent were used to generate the bone mask.

2. Bo-Net image preparation

Target masks were created through manual segmentation using ImageJ software [31,32]. Masks annotated on input images served as reference for the model training process. Training images were independently annotated by three trained annotators according to standardized annotation guidelines and quality-control procedures. The use of multiple annotators was intended to minimize individual annotation bias and improve the robustness and generalizability of the training dataset by incorporating inter-annotator variability. Prior to model training, all annotations were reviewed for consistency to ensure uniform labeling across the dataset. For the detection of a specific type of object, a two-class approach was employed. The first class represented the object of interest, i.e., pixels corresponding to OCs, OBs, bone, and vessels, and was assigned a value of 1 (white). The second class represented the background and was assigned a value of 0 (black). This labeling scheme enabled the neural network (NN) to effectively learn to differentiate between the object of interest and the background, facilitating the segmentation of the input image. The following steps were consistently applied to every cell structure dataset: train-validation split, patching, and data augmentation.

i Train-Validation split. The dataset was divided into 80% training and a 20% validation subset at the sample level; the same stack was never included in both sets. As mentioned in the paragraph ”sample staining and acquisition”, bone samples were sectioned into 300-µm-thick vibratome slices, such that even consecutive sections represented distinct tissue volumes and therefore provided biologically unique information. To avoid data leakage and ensure biological independence between training and validation data, the split was performed before patch extraction and data augmentation. Following split, patch extraction and data augmentation were performed independently within each subset. This procedure yielded a final dataset of 21,395 images, including 17,104 images for training and 4,291 images for validation. This process was accomplished using the Python scikit-learn library [33]. The training subset was utilized to train the model, while the validation subset served as an impartial evaluator of the model performance on unseen data. By incorporating these practices during the train-validation split and pre-processing stages, we ensured the robustness and effectiveness of the model while preserving its ability to handle novel data scenarios effectively.
ii Image2patch. To address the computational limitations posed by the original image sizes during the training phase, we applied a cropping approach, instead of resizing via interpolation, which could potentially alter crucial pixel values and affect the performance of the deep learning algorithm. We used an in-house developed tool, named “image2patch” available on PyPI (https://pypi.org/project/image2patch/), to avoid pixel loss recorded with classic patching techniques. The “image2patch” algorithm effectively minimizes pixel loss by dynamically adapting the patch stride to match the image original dimensions, thereby creating small overlaps. This mitigates information loss and acts as an augmentation process, enhancing the dataset’s diversity and bolstering the algorithm’s robustness. The ability to retrieve multiple patches from a single original image provides an extensive pool of data for the DL algorithm to learn from and make predictions. During the training phase, the patch size was consistently set at 512x512 pixels, ensuring a standardized and manageable format for the input data. To illustrate the effectiveness of the approach, examples are provided in S5 Fig, demonstrating how this strategy facilitates the effective utilization of large images, optimizes the learning process, and significantly contributes to the overall success of the DL model. Patches with <2.5% of white pixels, considered not informative, were removed from the pool not to lead to inaccurate predictions and suboptimal results.
iii Data Augmentation. To enhance model performance and increase dataset size, data augmentation was employed, including discrete rotations of π/2 and transposition. Elastic deformations were avoided to preserve the integrity of the cells’ shapes, as pertinent to the algorithm success. Additionally, a random pre-processing step between the two following options was incorporated and applied to the rotated versions of the images: either automatic histogram equalization (Contrast Limited Adaptive Histogram Equalization: CLAHE [34]) or custom pre-processing with varying values of gamma and threshold. These gamma and threshold values were chosen randomly within a specific range, to maintain the minimum visibility and quality of the images. This method was employed to avoid the risk of applying transformations that might result in images excessively dark or overly bright, making them unsuitable for processing by the neural network.

The choice of the pre-processing method in data augmentation was governed by a probability factor, ensuring diversity in the process. Via data augmentation, a wealth of new data points was generated, enriching the dataset with a wide range of pixel values and augmenting variability, enabling the NN to train on diverse image characteristics and become insensitive to fluctuations in brightness and contrast, which often result from variations in the image acquisition process.

We obtained a total of 21,395 processed images/patches, including 17,104 for training and 4,291 for validation, as reported in Table 2, which displays the final number of images for both the training and validation datasets.

**Table 2:** Number of processed images/patches after animal-level train-validation split, patch extraction, filtering, and data augmentation.

Cell type	Training	Validation
OB	3542	849
OC	3840	768
Bone MPM	3728	786
Bone CM	2904	1248
Vessels	3090	640

3. Model Training

i ***CNN Architecture.*** We applied a a customized version of the U-Net convolutional neural network [18,35] augmented with a ResNet34 [36] as the encoder. Models were trained using a local workstation equipped with an RTX A 5000 GPU and 128GB DDR4 RAM. We used Python libraries TensorFlow [37], Keras [38] and Segmentation Models [39] to build, train, and validate our models and applied classic statistical metrics such as precision, recall, and intersection over union (IoU) to evaluate models’ outcome during the test phase. In particular, IoU is commonly used to measure performance in object detection tasks, and it is calculated by dividing the intersection between the ground truth and the predicted mask by the union of the two. In formula:

$$\begin{array}{c} IoU=\;\frac{Area\;of\;Overlap}{Area\;of\;Union} \end{array}$$ (4)

$$\begin{array}{c} Accuracy=\;\frac{TP+TN}{TP+TN+FP+FN} \end{array}$$ (5)

$$\begin{array}{c} Precision=\;\frac{TP}{TP+FP} \end{array}$$ (6)

$$\begin{array}{c} Recall=\;\frac{TP}{TP+FN} \end{array}$$ (7)

ii Hyperparameters Tuning. Hyperparameter tuning enabled us to optimize the model performance for the specific task at hand. In our bone stromal cell segmentation task, we fine-tuned batch size, number of epochs, lambda regularization, number of filters and learning rate. For the latter, to strike a balance between fast convergence and stable training, we implemented a sigmoid-like learning rate function, reported in Eqs. (8) and (9).

$$\begin{array}{c} r(E)=\left\{ \begin{array}{c} @l\text{0}\;if\;x_{i,j}\;if\;E<E_{threshold}\\ ae^{-\frac{D_{i,j}}{\text{2}\sigma^{\text{2}}}}\;otherwise \end{array}\right. \end{array}$$ (8)

Where:


$$\begin{array}{c} s(E)=\;\frac{E-\;\frac{E_{threshold}}{\text{2}}}{\frac{E_{threshold}}{\text{8}}} \end{array}$$ (9)

To identify the most suitable hyperparameters for each bone stromal cell segmentation task, we conducted a randomized search across a predefined hyperparameter space by applying the Hyperband algorithm [40]. This approach enabled us to efficiently explore a broad range of hyperparameter values and pinpoint the optimal combination that yielded the highest segmentation accuracy. For each cell type, distinct hyperparameter values were carefully selected, considering the specific characteristics and challenges posed by each cell structure.

The resulting hyperparameter configurations are detailed in Table 3, reflecting the fine-tuned settings that maximize the segmentation performance for each cell type.

**Table 3:** Hyperparameter values. The Number of Filters is the number of convolution filters in the top layer.

Cell type	Learning Rate	Batch Size	Epochs	λ Regularization	Number of Filters
OB	0.0005	32	300	0.01	28
OC	0.001	16	300	0.0005	32
Bone MPM	0.00005	32	300	0.005	28
Bone CM	0.0001	24	300	0.001	32
Vessels	0.001	16	300	0.001	32

iii. Loss Function. We utilized the Tversky Loss (TL) [41] as the loss function for training our DL models. This loss function penalizes false positives (FPs) and false negatives (FNs) by incorporating weights for these errors, controlled by two parameters: alpha (α) and beta (β). Additionally, to address the challenge of class imbalance, we further weighted the loss function based on class-specific weights. The TL writes:

$$\begin{array}{c} TL_{c}=\sum_{c}\left(\text{1}-TI_{c}\right) \end{array}$$ (10)

With:


$$\begin{array}{c} TI_{c}=\;\frac{\sum_{i=\text{1}}^{N}p_{ic}g_{ic}+\epsilon }{\sum_{i=\text{1}}^{N}p_{ic}g_{ic}+\;\alpha \sum_{i=\text{1}}^{N}p_{i\overset{―}{c}}g_{ic}+\;\beta \sum_{i=\text{1}}^{N}p_{ic}g_{i\overset{―}{c}}+\epsilon } \end{array}$$ (11)

where the TL for a certain class c (representing a specific cell type) is calculated based on the Tversky index (TI). *p*_*ic*_ is the probability of the i-th pixel to be of class *c*, while *g*_*ic*_ represents the ground truth value for that pixel. In the same way $p_{i\overset{―}{c}}$ and $g_{i\overset{―}{c}}$ refer to the probability and ground truth of the background class. N is the total number of pixels in the image. The parameters α and β control the relative weighting of false positives and false negatives in the loss function. This function penalizes false positives and false negatives differently depending on the values of α and β.

To optimize the TL for our task, we empirically selected appropriate values for α and β, as presented in Table 4. These parameter values ensure the TL aligns with the specific challenges posed by each cell type and aids in achieving highly accurate segmentation results during the training process. Finally, the TL was minimized by Adam Optimizer [42].

**Table 4:** *α* and *β* chosen for the Tversky Loss in every trained model. The cell-types represent the dataset of cells for which the models were trained.

Training Model	α	β
OB	0.3	0.7
OC	0.3	0.7
Bone MPM	0.3	0.7
Bone CM	0.3	0.7
Vessels	0.7	0.3

4. Post Processing

In the post-processing phase, parameter extraction was performed in a Python environment, employing both custom functions and built-in libraries to retrieve crucial measurements for each cell type.

For OC analysis we extracted the following measurements: i) cell count (n) – we identified and counted OCs as 8-connected structures whose area exceeded a predefined threshold, classifying them as cells; ii) location (x,y) – to determine the location of each OC, we calculated the center of mass of the region, obtaining coordinates along the X and Y directions;

Due to the unique line-like appearance of OBs along the bone structure contour, the extraction of the same parameters as done for the OCs was not feasible. Consequently, we focused on calculating bone coverage as a key parameter for OB analysis. Bone coverage was determined by quantifying the percentage of the common area shared by the OBs and the bone contour, relative to the area of the bone contour alone. S6 Fig displays a visual representation of the process. This calculation is expressed in Eq. (12).


$$\begin{array}{c} Cell\;Bone\;Coverage=\;\frac{Common\;Area}{Bone\;Contour}\;(\%) \end{array}$$ (12)

To address the imperfect overlap between the bone contour and the OB prediction, which resulted in the underestimation of the coverage, a morphological transformation called dilation was employed. The purpose of this dilation operation was to increase the width of the bone contour, enhancing the accuracy of bone coverage calculation. The process is described by Eq. (13):


$$\begin{array}{c} A⨁B=\;{⋃}_{b\subset B}A_{b} \end{array}$$ (13)

In it, A represents the bone contour, and B is a 3x3 structuring element used in the dilation process. The dilation operation enlarges the bone contour by considering the intersection of the structuring element B with the bone contour A. As a result, the contour width increases, effectively compensating for any misalignment between the bone contour and the OB prediction. OC predictions might be affected by false positives in regions with bright cells that are not OCs. To overcome this limitation, OC bone coverage was calculated following the same algorithm and calculations.

## Results

### Preclinical mouse modeling and image acquisition

**Fig 1:**
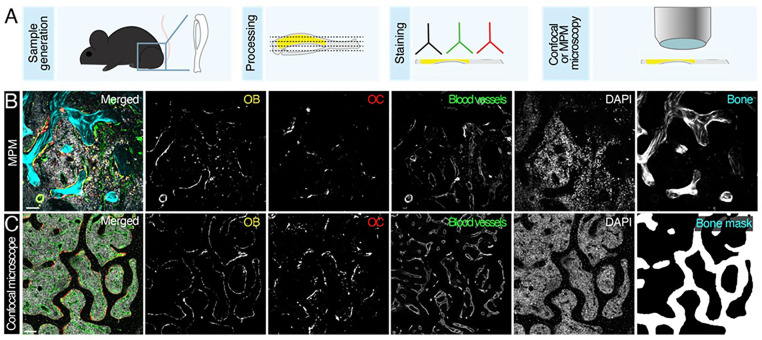
Generation of samples by multiphoton and confocal microscopy. **A.** Schematic representation of the experiment. **B, C**. Imaging of bone samples. Merged and single-channel pictures are shown. OB (alkaline phosphatase); OC (TRAP); blood vessels (endomucin); DAPI (total nuclei); bone and bone mask. Scale bar: 100 μm.

To monitor bone biology by confocal or multiphoton microscopy, we established a pipeline for image analysis based on ex vivo processing and staining of mouse bones. Processing consisted of tissue fixation, decalcification, and generation of 3D bone slices (300 µm thick) at the vibratome, followed by staining and multiparametric acquisition of 3D stacks by confocal microscopy (CM: 85 stacks) or multiphoton microscopy (MPM; 90 stacks), for a total of 5,530 single images (Fig 1A). Specifically, we detected OBs (alkaline phosphatase), OCs (tartrate-resistant acid phosphatase, TRAP), blood vessels (endomucin; Fig 1B, C), and total nuclei (4′,6-diamidino-2-phenylindole, DAPI) for internal reference. MPM allowed the acquisition of collagenous matrix in bone through the detection of second harmonic generation (SHG) signal. SHG is a label-free, non-linear function of light resulting from frequency doubling of photons that interact with non-centrosymmetric molecules and materials, including coiled-coil or polymeric proteins (such as fibrillar collagen; Fig 1C). CM does not allow acquisition of label-free bone signal. To overcome this issue and include bone information in our analyses, we superimposed the fluorescent signal of all the channels acquired (including DAPI) and generated a mask of acellular areas (Fig 1C). Images from different channels were annotated under the supervision of expert biologists, to generate ground-truth labels for each parameter for training and performance evaluation.

### Model performance: Training and validation

**Fig 2:**
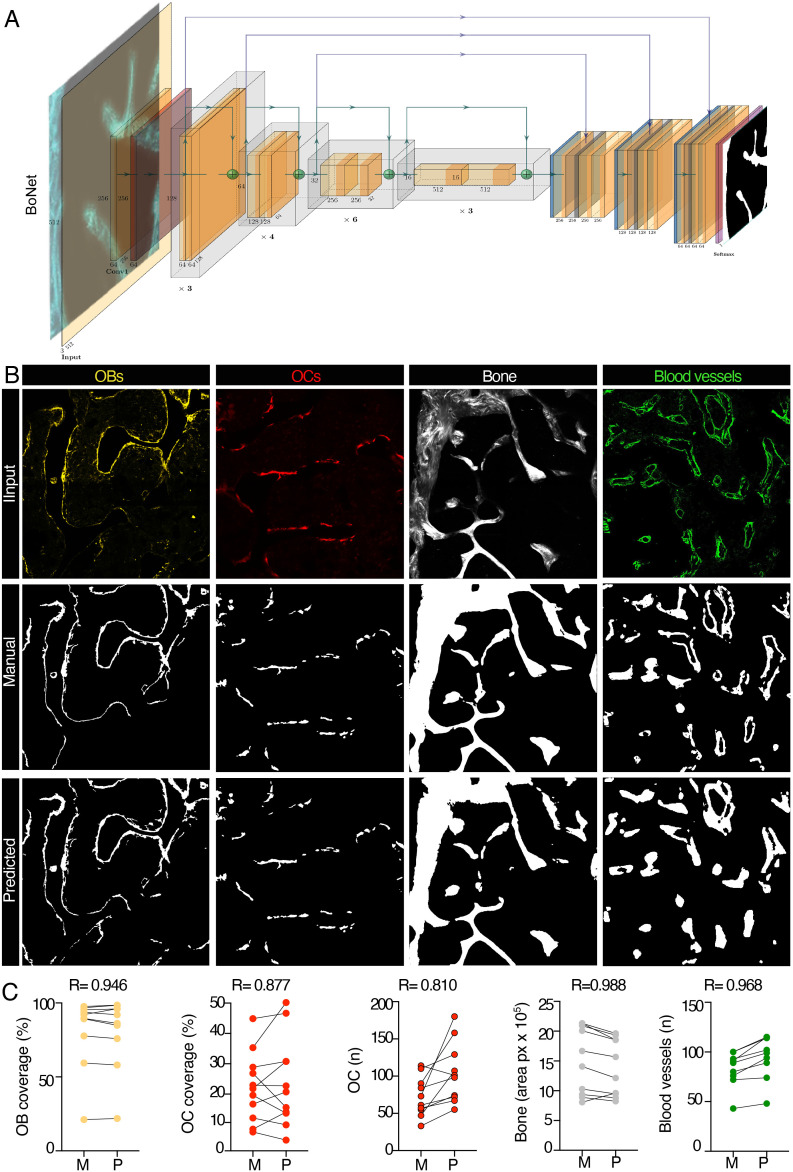
A. Model performance. Fully Convolutional Network scheme. Data flows from left to right through convolutional layers. **B**, **C**. Input, manual output, and predicted output are shown (B) together with Pearson correlation between manual and predicted outputs (obtained from 8-10 independent images that were not included in the training or validation sets) (C).

To determine the optimal infrastructure for our segmentation task, we evaluated four neural networks: a customized UNet adapted from our group and previously applied to the study of the foreign body response [18,35], UNet++, ResNet, and UNet augmented with ResNet [35,43,44]. Our goal was to identify the best configuration for segmenting OCs, which presented the greatest challenge, and subsequently apply it to segment the remaining cellular and extracellular structures of interest. All neural networks were trained using the Tversky Loss function with hyperparameters α = 0.3 and β = 0.7. Based on superior Intersection over Union (IoU) scores (metrics detailed in the Methods section) across both training and validation sets (see Table 5), we selected the UNet augmented with ResNet34 as the encoder (Fig 2A). As an advantage, this model further offered high effectiveness, minimal computational demands, and effectively captured the similarity in input images captured by microscopy.

**Table 5:** Intersection over Union (IoU) values during training and validation for neural network configurations evaluated for OC segmentation.

Neural Network	Training IoU	Validation IoU
Customized UNet	0.6927	0.4825
UNet++	0.6995	0.464
ResNet	0.4813	0.3912
UNet – ResNet34	0.7147	0.5728

Once selected the optimal neural network, the training phase was carried out with the dual objectives of minimizing the loss function and simultaneously maximizing the Intersection over Union (IoU) metric (both presented in the Methods section).

Starting from the original dataset, images were first split at the animal level into training and validation subsets, as described in the Materials and Methods. Patch extraction, preprocessing, filtering, and data augmentation were then performed independently within each subset, resulting in a total of 21,395 processed images/patches: 17,104 for training and 4,291 for validation distributed as follows: OBs (3542 images for training, 849 for validation), OCs (3840 images for training, 768 for validation), vessels (3090 images for training, 640 for validation), and bone structures both acquired at MPM (3728 images for training, 786 for validation), and CM (2904 images for training, 1248 images for validation; Table 2, in the Materials and Methods).

To meet the optimal trade-off between model performance and temporal demands for the training process, the training spanned 300 epochs. The resulting metrics for the training and validation set are fully presented in Table 6, while the loss function minimization and IoU maximization against the epochs are plotted in S7 Fig.

**Table 6:** IoU values over the training and validation phases.

Cell Type	Training IoU	Validation IoU
OB	0.8345	0.5517
OC	0.7147	0.5728
Bone MPM	0.8204	0.7651
Bone CM	0.8084	0.7118
Vessels	0.6191	0.4244

The IoU was evaluated on the validation set to verify model generalization capability. The model achieved substantial IoU scores during the training phase, showing a high learning capacity, and high capability in recognizing different cellular patterns. OBs, bone acquired by MPM and bone mask from CM (characterized by relatively well-defined and consistent shape) were associated with higher IoU scores (0.83, 0.82, and 0.81 respectively) than OCs and vessels (0.72 and 0.62 respectively; Table 6, S3 Fig) that exhibited instead more intricate and variable structures. Further, we conducted an additional performance evaluation using a distinct dataset not employed in prior training or validation phases (test set; n = 8–10 images/object class). Given the absence of automated tools for detecting and quantifying the targeted cellular structures, the evaluation relied on assessing the disparities between the model’s predictions and ground truth segmentation masks generated manually by an operator.

We observed a notable disparity between the IoU performance on training and validation sets for OBs (0.8345 vs. 0.5517), OCs (0.7147 vs. 0.5728), and vessels (0.6191 vs. 0.4244), indicating potential overfitting to these structures and suggesting limited generalization to new, unseen data. To address overfitting and enhance model generalization in future applications, increasing both the quantity and quality of the dataset is recommended.

To this purpose, we employed a comprehensive set of pixel-wise evaluation metrics, such as precision, recall, and IoU (Table 7). Precision, representing the model ability to avoid false positives, showed heterogeneous values across the cellular structures, with bone achieving higher precision (0.99 and 0.92 for CM and MPM, respectively) than OBs, OCs, and vessels (0.77, 0.78, and 0.76 respectively). Recall, measuring the proportion of true positives among all the actual positives, achieved values ranging from 0.77 (vessels) to 0.81 (OCs). Finally, the IoU, measuring the overlap between the predicted and ground truth masks ranged from 0.6 (vessels) to 0.9 (bone CM). As expected, due to their consistent shape (low intra-variability), high contrast with the background, and abundance in the dataset, bone structures exhibited a higher IoU score, being the most important metric in image segmentation, compared to OBs, OCs, and vessels. These latter are indeed characterized by intricate shapes with high intra-variability, lower contrast against the background, and lower prevalence in the dataset. To further validate the performance of our automatic segmentation tool, we compared the predicted bone contour coverage by OBs and OCs (S4 Fig), the OC and blood vessel count, and bone area against the manually segmented test set of images (Fig 2B, C). The bone area quantified by Bo-Net was comparable to manual measurements, with a Pearson correlation coefficient of R = 0.988; for the bone contour of OBs and OCs, the score was R = 0.946 and R = 0.877, respectively. The OC and blood vessel number showed a score of R = 0.810 and R = 0.968, respectively (Fig 2C). These results suggest that Bo-Net can achieve segmentation accuracy comparable to experienced biologists (R = 0.81–0.988 for the parameters tested) while improving analysis time from days to seconds.

**Table 7:** Average pixel-wise metrics for the different biological structures of interest in the test dataset. Calculated from n = 8-10 manually annotated images per object class.

Cell Type	Precision	Recall	IoU
OB	0. 7659	0. 7990	0. 6375
OC	0. 7806	0. 8069	0. 6603
Bone MPM	0. 9208	0. 8847	0. 8215
Bone CM	0. 9899	0. 8950	0. 9019
Vessels	0. 7626	0. 7681	0. 6003

### Segmentation and quantification of OB and OC markers by Bo-Net

To validate the biological relevance of our segmentation approach, we performed a series of experiments and quantified outcomes with our Bo-Net. In the first experiment, we applied Bo-Net and compared the percentage of OB and OC bone coverage in immunofluorescence analysis performed on genetically engineered fluorescent reporter mouse models vs antibody-based staining.

**Fig 3:**
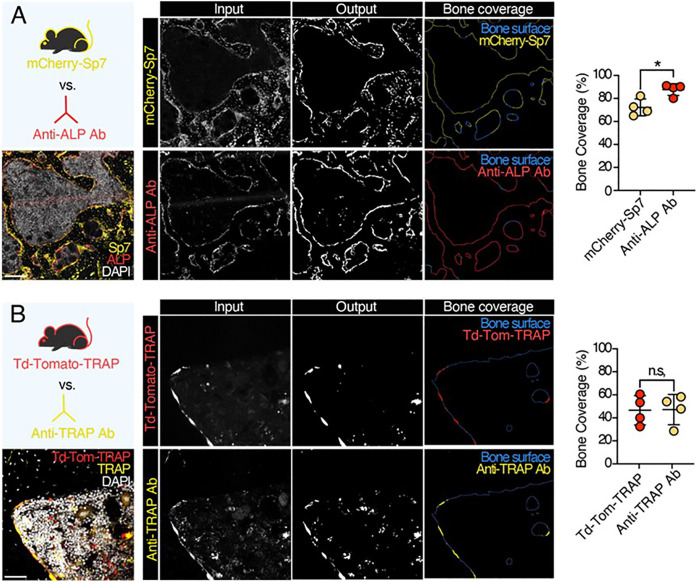
Automatic quantification of reporter mouse- vs. antibody-based detection of OBs and OCs. **A.** Schematic representation of the markers detected;. mCherry-Sp7, endogenous fluorescence; goat anti-ALP Ab + donkey ant-goat AlexaFluor 680. Representative input (merged and single channels), output and bone coverage images and a quantification of bone coverage (%) are shown; n = 4 tibiae/group, 1 max projection/tibia; images were acquired at the confocal microscope. **B.** Schematic representation of the markers detected;. Td-Tomato-TRAP, endogenous fluorescence; rabbit anti-TRAP Ab + donkey ant-rabbit AlexaFluor 680. Representative input (merged and single channels), output and bone coverage images and a quantification of bone coverage (%) are shown; n = 4 tibiae/group, 1 max projection/tibia; images were acquired at the confocal microscope. *p < 0.05; n.s., non-significant; unpaired two-tailed Student t-test. Scale bar, 100 μm.

To visualize OBs, we used mCherry-Sp7^+^ mice (which expressed fluorescently labeled transcriptional factor osterix) [45] or an antibody against alkaline phosphatase (ALP), a membrane-bound glycoprotein produced during bone mineralization (Fig 3A). Bo-Net segmented and quantified 15% less signal in mCherry-Sp7^+^ samples compared to anti-ALP antibody (Fig 3A). This result was expected, given the localization of these two proteins, which is mostly confined in the nucleus for osterix while cytoplasmic/cell membrane bound for ALP. Interestingly, osterix was further expressed by osteocytes, the bone cells formed when OBs are embedded in the calcified matrix they secreted. However, the presence of a positive signal of osteocytes within bone did not impact the ability of Bo-Net to quantify OBs at the bone interface (Fig 3A). Comparison with manual quantification demonstrated moderate to strong correlations for both osteoblast markers (S8A Fig.), supporting the ability of Bo-Net to capture biologically relevant differences despite distinct labeling patterns.

OCs were detected through visualization of TRAP, a glycosylated monomeric metalloprotein enzyme involved in bone resorption, expressed by Td-Tomato-TRAP^+^ mice [46] vs anti-TRAP antibody (Fig 3B). Bo-Net segmentation of TRAP^+^ mouse-derived tissue vs staining, instead, did not show any significant difference (Fig 3B). Manual and automated quantification also showed strong correlations for osteoclast markers (S8A Fig.). These results suggest that Bo-Net could segment and quantify different markers in a marker-specific fashion. If the same marker was detected with different methods (i.e., fluorescent reporter mouse vs antibody), this did not significantly impact the segmentation and quantification process.

### Segmentation and quantification of the impact of intrinsic and extrinsic modifications of OBs and OCs by Bo-Net

**Fig 4:**
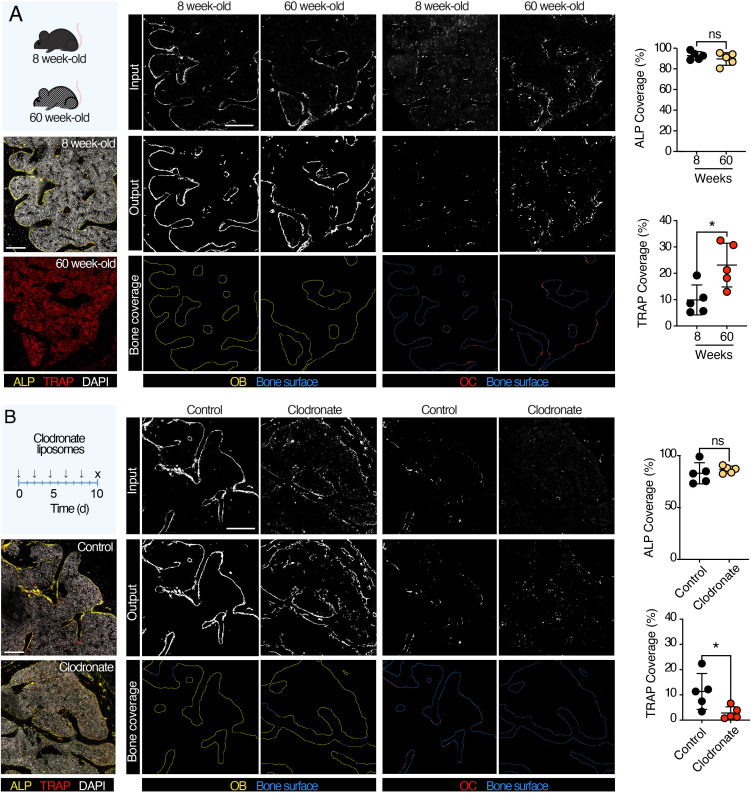
Automatic quantification of OBs and OCs in mice of different ages and after clodronate treatment. **A.** Schematic representation of the experiment; representative input (merged and single channels), output and bone coverage images for 8 and 60-week-old mice; a quantification of ALP and TRAP coverage (%) is shown; n = 5 tibiae/group, 1 max projection/tibia; images were acquired at the confocal microscope. Goat anti-ALP Ab + donkey ant-goat AlexaFluor 594 and rabbit anti-TRAP Ab + donkey ant-rabbit AlexaFluor 680 were used. *p < 0.05; n.s., non-significant; unpaired two-tailed Student t-test. Scale bar, 100 μm. **B.** Schematic representation of the treatment schedule; representative input (merged and single channels), output and bone coverage images; a quantification of bone coverage (%) is shown; n = 5 tibiae/group, 1 max projection/tibia; images were acquired at the confocal microscope. Goat anti-ALP Ab + donkey ant-goat AlexaFluor 594 and rabbit anti-TRAP Ab + donkey ant-rabbit AlexaFluor 680 were used. *p < 0.05; n.s., non-significant; unpaired two-tailed Student t-test. Scale bar, 100 μm.

As a further biological validation of Bo-Net for OBs and OCs segmentation and quantification, we performed analysis of bone samples derived from mice at different age. Aging is associated with increased osteoclastogenesis, increased bone resorption, and consequent loss of bone mass [47]. We compared the expression of ALP (OBs) and TRAP (OCs) in mouse tibiae from 8 weeks- vs 60 week-old (Fig 4A). Bo-Net did not identify a significant impact of aging on OB coverage of bone, however it detected a significant increase in the OCs coverage (almost double the amount; Fig 4A), in line with published evidence [47].

These results were supported by manual quantification, which showed good agreement with automated OB measurements and very strong agreement for OC measurements (S8B Fig). In another set of mice, we performed pharmacological treatment of 8 week-old mice with liposomal clodronate, a bisphosphonate that upon internalization by phagocytes, including macrophages and osteoclast precursors, induces their apoptosis [48]. Mice were treated 5 times, once every 2 days, sacrificed, and bone stained for ALP and TRAP (Fig 4B). Clodronate liposomes did not affect OB coverage of bone (values remained stable at around 90% of bone coverage in both control and treated mice), whereas they significantly reduced the number of OCs (average bone coverage decreased of ~50% in treated samples; Fig 4B). Manual and automated quantification were again in strong agreement (S8C Fig). These results suggest that Bo-Net could detect and quantify the impact of intrinsic (aging) or extrinsic (pharmacological targeting) processes that impact OBs and OCs.

### Segmentation of tumor-induced OCs by Bo-Net

**Fig 5:**
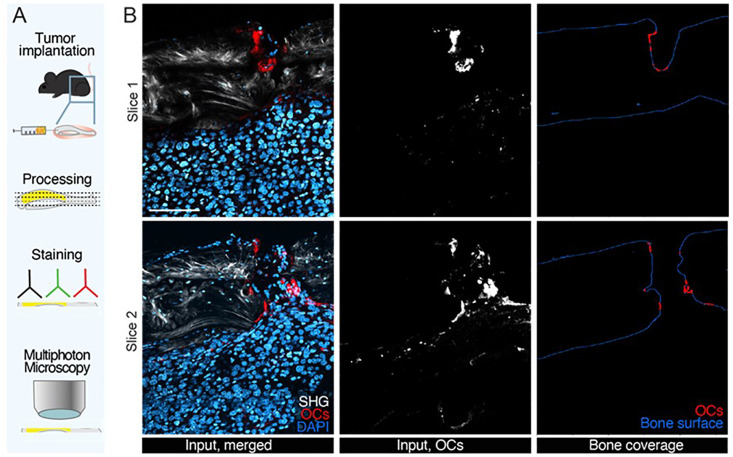
Automatic detection of tumor-induced OCs. **A.** Schematic representation of the experimental pipeline; **B**. Representative input (merged and single channel), and bone coverage images. Scale bar: 100 μm.

As the next step, we applied Bo-Net to detect tumor-induced osteolysis. Renal cancer bone metastasis are known to induce recruitment and activation of OCs in patients [49,50], followed by pathologic bone resorption, which results in skeletal-related complications including pain, hypercalcemia, spinal cord compression, fractures, and limited mobility [49,50]. To monitor tumor-induced bone resorption, RENCA renal tumor cells were implanted in mouse tibiae and samples were processed for microscopy-based analysis (**Fig 5A).** These tumor cells induced OC recruitment, which was successfully segmented by Bo-Net, revealing areas of pathologic bone resorption and disruption of the mineralized bone structure (**Fig 5B**).

### Segmentation and quantification of bone and tumor-induced blood vessels by Bo-Net

**Fig 6:**
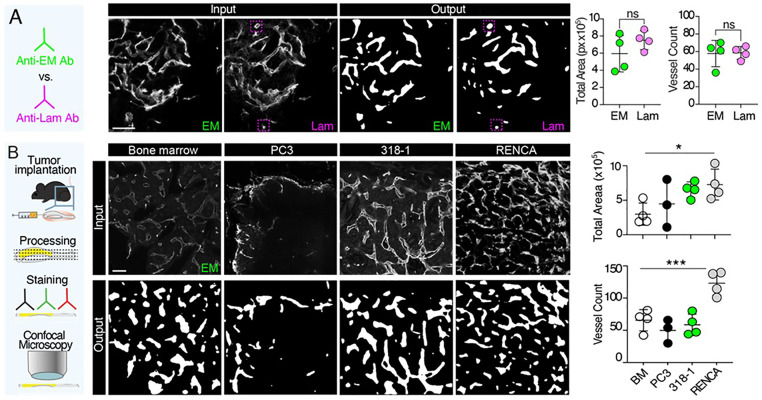
Automatic quantification of antibody-based detection of blood vessels. **A.** Schematic representation of the markers detected; representative input and output images; a quantification of total vessel area and vessel count is shown. n = 4 tibiae/group, 1 max projection/tibia; images were acquired at the confocal microscope. Rabbit anti-Lam Ab + donkey ant-rabbit AlexaFluor 680 and donkey ant-goat AlexaFluor 594 and rat anti EM Ab + anti-rat AlexaFluor 488 were used; n.s., non-significant, unpaired two-tailed Student t-test. Scale bar, 100 μm. **B.** Schematic representation of experimental pipeline; representative input and output images; a quantification of total vessel area and vessel count is shown; n = 3-4 tibiae/group, 1 max projection/tibia; images were acquired at the confocal microscope. Rabbit anti-Lam Ab + donkey ant-rabbit AlexaFluor 680 and donkey ant-goat AlexaFluor 594 and rat anti EM Ab + anti-rat AlexaFluor 488 were used; *p < 0.05; *** p < 0.001 unpaired two-tailed Student t-test.

We then biologically validated the ability of Bo-Net to segment and quantify blood vessels in bone. Mice were implanted with different tumor cell lines: PC3 human prostate cancer cells, 318−1 murine osteosarcoma cells, and RENCA murine renal cancer cells. Bones were processed and stained for endomucin and/or laminin, two proteins expressed in bone sinusoidal blood vessels or in the blood vessel basal membrane, respectively. Then, images were acquired at the confocal microscope (Fig 6A, B) and analyzed. Vessel number was quantified by counting segmented structures after applying a minimum size threshold of 10 × 10 pixels (≈5.5 × 5.5 µm) to exclude artifacts and background noise. When directly comparing endomucin and laminin expression in RENCA tumors, Bo-Net did not identify significant differences in the number and area of blood vessels. Besides staining sinusoidal blood vessels, the anti-laminin antibody further recognized bone arteries/arterioles, which represent a limited number of bone blood vessels (Fig 6A, dotted box), and did not significantly impact the total area or vessel number, as expected. Again, manual analyses were in strong agreement (S8D Fig). When applying Bo-Net to quantify the vasculature induced by different tumors in bone vs tumor-free bone marrow, RENCA renal cells, which are known to be particularly angiogenic, showed a significant increase in the total blood vessel area and vessel count, while 318−1 and PC3 tumors did not show a significant increase (Fig 6B).

These results suggest that Bo-Net could detect and quantify bone marrow and tumor-induced blood vessels in bone.

## Discussion

We developed Bo-Net, a software tool for automated multiparametric analysis of fluorescent microscopy images of cellular (OBs, OCs, and endothelial cells) and extracellular (bone matrix) bone components. Bo-Net is free and available to the bone research community. Additionally, we created an executable software that includes a user-friendly graphic user interface integrating our deep learning-based models. The software was designed to be accessible by scientists who may have limited programming knowledge, offering an easy-to-use tool for cell segmentation and post-processing. Access to high-performance hardware (such as GPUs or TPUs) was essential to guarantee efficient model training, but Bo-Net can operate on any computer, without specific requirements.

Bo-Net relies on deep learning to perform semantic segmentation, extraction of parameters, and quantification of structures of interest from the same image. Our automated tool took just a few seconds to perform analysis that would take hours when performed manually. Currently available general-purpose image segmentation algorithm do not directly perform differential segmentation and quantification of multiple bone stromal cells and bone within the same fluorescence microscopy image. In line with this, we evaluated the applicability of StarDist and Cellpose, two widely used tools, to representative images from our dataset. StarDist was not well suited to our images, which predominantly contain flat, linear structures or irregularly shaped objects rather than round cells or nuclei. We also tested Cellpose using representative images of OB and BV, and even after several optimization attempts involving the adjustment of input parameters, the segmentation quality remained suboptimal. In Sp7 images, where the marker is expressed by both OBs and osteocytes, Cellpose preferentially segmented osteocytes rather than OBs lining the bone surface, which were the cells of interest. Similarly, for BV analysis, repeated parameter optimization did not substantially improve vessel recognition. These observations suggest that, despite their broad utility, currently available generalist tools have limited applicability regarding the specific morphologies detected in this study, further supporting the utility of Bo-Net. As a further advantage, Bo-Net could process images acquired at multiphoton or confocal microscopes, which are acquired with different objectives, at different resolutions and with different sizes. We acknowledge that imaging conditions may vary between laboratories due to differences in microscope systems, acquisition settings, staining protocols, and sample preparation. Bo-Net was trained using a large dataset based on acquisition with a confocal and a multiphoton microscope, which improved its robustness to such variability. We anticipate that the pretrained model will generalize well to datasets acquired under similar imaging conditions. Nevertheless, for substantially different imaging setups or staining protocols, additional user-generated annotations and model fine-tuning may further optimize performance.

We adopted a U-Net model, augmented with a ResNet-34 as the backbone. U-Net is particularly suitable in image segmentation tasks due to its architecture, which combines an encoding and a decoding path and allows complex features to be captured while preserving spatial information. The choice of ResNet-34 as backbone was dictated by its ability to train complex neural networks mitigating the vanishing gradient problem (decrease in loss function minimization efficiency during training). We conducted extensive training of 300 epochs, which is a reasonable compromise between training accuracy and computational demand. In detail, we initially employed an Early Stopping approach, which involves terminating the training phase when the model’s performance on the validation set begins to decline to prevent overfitting. This strategy limited training to a maximum of 30 epochs, resulting in unsatisfying metrics. Consequently, we adopted a gradual increase in the number of epochs while monitoring the divergence in loss function minimization between the training and validation sets.

We assessed model performance at two levels. During model development, training and validation performance were monitored using IoU. Further, an independent manually annotated test dataset, not used for training or validation, was used to calculate pixel-wise precision, recall, and IoU.The training performance spanned a large spectrum across different cell and tissue structures, according to IoU analysis. OBs and bone were associated with higher IoU than OCs and blood vessels, suggesting different recognition capability for different bone components. These variations in performance highlighted the model’s strengths in segmenting certain structures, such as OBs and bone, while suggesting room for improvement in OCs and mostly blood vessel segmentation. This difference was likely due to the intrinsic structural complexity of blood vessels and OCs, while OBs and bone exhibit relatively well-defined and consistent shapes, which made them easier to be accurately segment. We also recorded a slight discrepancy between training and validation IoU performance for OBs, OCs, and vessels, indicating a degree of overfitting for these structures, and suggesting that the model may not generalize well to new, unseen data. This was also confirmed by comparing training and validation IoU trends, and it further highlighted better performance related to bone structures due to their easily recognizable features and low inter-variability. Boosting both the quantity and quality of the dataset might mitigate overfitting and enhancing model generalization in future developments.

We derived a series of parameters, such as bone area, bone contour coverage, cell/blood vessel count and validated our model against images manually annotated by experts providing a ground truth for performance assessment. Bone area quantification identified pixels belonging to bone structures, with high performances. The bone contour coverage quantified the proportion of OBs or OCs situated along the bone contour. These analyses focused on regions of interest and disregarded non-interesting regions, like bone marrow or osteocytes. Despite the segmentation challenges posed by irregular OB and OC shapes, the model performed well in this regard, as evidenced by the high correlation (R = 0.946 for OBs and R = 0.877 for OCs). OC/blood vessel count evaluation was based on two criteria: interconnectedness and proximity to ground truth centroids. These criteria were essential to determine whether clusters of white pixels qualified as structure of interest and, again, showed challenges in automatic segmenting OCs and blood vessels, resulting in moderate performance with an error percentage of 14.4% and 13.6%, respectively. Our comparative analyses demonstrate that Bo-Net is robust across different staining strategies and imaging conditions when markers label the same biological structures. Specifically, no significant differences were observed between fluorescent reporter- and antibody-based TRAP labeling, or between endomucin and laminin staining for vascular analysis, despite differences in signal intensity and background levels. In contrast, discrepancies observed between alkaline phosphatase and Sp7 labeling likely reflect intrinsic biological differences between these osteoblast markers, including their distinct cellular localization and expression patterns. Together, these findings indicate that segmentation performance is primarily influenced by marker biology rather than technical variations in staining approach, supporting the adaptability of Bo-Net across diverse experimental settings.

From a biological standpoint, Bo-Net satisfyingly segmented and quantified OBs, OCs and blood vessels on confocal and multiphoton microscopy images captured in different conditions, spanning therapeutic treatment, bone resorption, tumor angiogenesis in bone and impact of aging on bone cells.

We purposely perturbed the bone system with stimuli that have a known outcome (e.g., administration of clodronate liposomes is known to reduce the number of OCs; tumor implantation induces formation of OCs and lead to neoangiogenic blood vessels) or compared the impact of aging in young adult vs aged mice (which is associated to increased recruitment of OCs). Bo-Net correctly segmented and quantified these images, showing high reliability for analysis of unknown phenomena that can affect OB, OC and blood vessel biology in bone. Furthermore, Bo-Net correctly segmented images deriving from fluorescence reporter mice and antibody-based staining, demonstrating both flexibility and reliably.

In conclusion, Bo-Net is an innovative application of deep learning in the context of bone tissue image analysis that can help deepen the understanding of bone biology at the cellular and tissue levels, with broad implications in the field of space biology investigations. While we acknowledge the use of established architectures and no introduction of new deep learning models, we believe our work makes significant contributions in multiple perspectives, as follows. (i) Methodological contribution: Although we employed well-known architectures, our contribution highlights the adaptability of pre-existing models for segmenting complex biological structures and the ability to build on robust available tools to have a real impact in a wet lab environment. Technically, we highlighted the importance of effective model selection by comparing the performance of 4 different neural networks and application of objective criteria, configurations, parameter tuning and validation strategies tailored for biological image analysis, which are critical to support automation and reproducibility when addressing specific preclinical questions.(ii) Biological significance: The application of deep learning for analysis of biomedical images is relatively novel but in continuous expansion and it is crucial for advancing research. Our study addressed a significant challenge in bone/bone stromal cell segmentation, which has implications for understanding bone biology, pathology, and potentially therapeutic interventions. As a result, our work will make an AI-based tool readily available to the community of cell and bone biologists, pathologists, and medical scientists. (iii)Translational impact: While we recognize the importance of technically advancing neural networks’ infrastructure, we also believe that effectively applying and translating existing models is equally valuable. Our work is an example of translating deep learning techniques into practical applications, leading to the automation of labor-intensive tasks, typically subject to human error, and improved image analysis efficiency. (iv) Interdisciplinary relevance: Our target included professionals interested in both the technical aspects of deep learning and its applications in various domains, such as biology and medicine. We believe our work provided valuable insights into how deep learning can be effectively applied to address biological questions and challenges, with the objective of reaching a diverse audience.

## Supporting information

S1 Fig Summary diagram of the computational workflow. (JPEG)

S2 Fig Pixel normalization process. (JPG)

S3 Fig Histogram Stretching process. (JPG)

S4 Fig Pixel weighting. (JPG)

S5 Fig Image patching using Image2patch. (JPG)

S6 Fig OB Bone Coverage. (JPG)

S7 Fig Loss function minimization and Intersection over Union (IoU) maximization against the epochs. (JPG)

S8 Fig Manual analysis and correlation analysis with Bo-Net. (JPG)

S9 Fig Graphical Abstract. (JPG)
