# Supplementary material for: Bo-Net: Deep learning-based model for automatic bone stromal cell segmentation of fluorescence microscopy images
Source: PLoS One. 2026 Aug 4;21(8):e0353796. doi: 10.1371/journal.pone.0353796 (PMC13436720; doi:10.1371/journal.pone.0353796)
Supplement: Supplementary file 1 [file pone.0353796.s011.docx]

Reply to the Editor

Journal Requirements:

When submitting your revision, we need you to address these additional requirements.

1. Please ensure that your manuscript meets PLOS ONE's style requirements, including those for file naming. The PLOS ONE style templates can be found at
   <https://journals.plos.org/plosone/s/file?id=wjVg/PLOSOne_formatting_sample_main_body.pdf>
   and
   <https://journals.plos.org/plosone/s/file?id=ba62/PLOSOne_formatting_sample_title_authors_affiliations.pdf>

We updated our manuscript to meet the PLOS ONE’s style requirements.

2. Please note that PLOS One has specific guidelines on code sharing for submissions in which author-generated code underpins the findings in the manuscript. In these cases, all author-generated code must be made available without restrictions upon publication of the work. Please review our guidelines at
   https://journals.plos.org/plosone/s/materials-and-software-sharing#loc-sharing-code
   and ensure that your code is shared in a way that follows best practice and facilitates reproducibility and reuse.

The code has been made publicly available on Github at: <https://github.com/scasarin87/Bo-Net>

3. Thank you for stating the following in the Acknowledgments Section of your manuscript:

"Animal studies were approved by the Institutional Animal Care and Use Committee of the University of Texas, MD Anderson Cancer Center, which is accredited by the Association for Assessment and Accreditation of Laboratory Animal Care. We thank Dr. Masaru Ishii for sharing TRAP-tD-tomato mice (Osaka University, Osaka, Japan). We thank Dr. Ralph Adams (Max Planck Institute for Molecular Biomedicine, Münster, Germany) for sharing mCherry-Sp7 mice. This work was supported by the Cancer Prevention and Research Institute of Texas (RP230160), the National Institutes of Health (R21 CA267312-01A1), the David H. Koch Center for Applied Research of Genitourinary Cancers, The University of Texas MD Anderson Cancer Center, and the John F. Jr. and Carolyn Bookout Presidential Distinguished Chair fund. The Genitourinary Cancers Program of the CCSG shared resources at The University of Texas MD Anderson Cancer Center was supported by the National Cancer Institute at the National Institutes of Health (P30 CA016672). The funders had no role in study design, data collection and analysis, decision to publish, or preparation of the manuscript."

We note that you have provided additional information within the Acknowledgements Section that is not currently declared in your Funding Statement. Please note that funding information should not appear in the Acknowledgments section or other areas of your manuscript. We will only publish funding information present in the Funding Statement section of the online submission form.

Please remove any funding-related text from the manuscript and let us know how you would like to update your Funding Statement. Currently, your Funding Statement reads as follows:

"This work was supported by the Cancer Prevention and Research Institute of Texas (RP230160), the National Institutes of Health (R21 CA267312-01A1), the David H. Koch Center for Applied Research of Genitourinary Cancers, The University of Texas MD Anderson Cancer Center, and the John F. Jr. and Carolyn Bookout Presidential Distinguished Chair fund. The Genitourinary Cancers Program of the CCSG shared resources at The University of Texas MD Anderson Cancer Center was supported by the National Cancer Institute at the National Institutes of Health (P30 CA016672)."

Please include your amended statements within your cover letter; we will change the online submission form on your behalf.

We removed the funding information from the acknowledgments; the funding statement reported is correct.

4. Thank you for stating the following financial disclosure:

"This work was supported by the Cancer Prevention and Research Institute of Texas (RP230160), the National Institutes of Health (R21 CA267312-01A1), the David H. Koch Center for Applied Research of Genitourinary Cancers, The University of Texas MD Anderson Cancer Center, and the John F. Jr. and Carolyn Bookout Presidential Distinguished Chair fund. The Genitourinary Cancers Program of the CCSG shared resources at The University of Texas MD Anderson Cancer Center was supported by the National Cancer Institute at the National Institutes of Health (P30 CA016672)."

Please state what role the funders took in the study. If the funders had no role, please state:

"The funders had no role in study design, data collection and analysis, decision to publish, or preparation of the manuscript."

If this statement is not correct you must amend it as needed.

Please include this amended Role of Funder statement in your cover letter; we will change the online submission form on your behalf.

The funders had no role in study design, data collection and analysis, decision to publish, or preparation of the manuscript. We have included this amendment in the cover letter that will be uploaded for re-submission.

5. Your ethics statement should only appear in the Methods section of your manuscript. If your ethics statement is written in any section besides the Methods, please delete it from any other section.

They now only appear in the Methods section.

6. We note that you have indicated that there are restrictions to data sharing for this study. PLOS only allows data to be available upon request if there are legal or ethical restrictions on sharing data publicly. For more information on unacceptable data access restrictions, please see
   <http://journals.plos.org/plosone/s/data-availability#loc-unacceptable-data-access-restrictions>

Before we proceed with your manuscript, please address the following prompts:

a) If there are ethical or legal restrictions on sharing a de-identified data set, please explain them in detail (e.g., data contain potentially identifying or sensitive patient information, data are owned by a third-party organization, etc.) and who has imposed them (e.g., a Research Ethics Committee or Institutional Review Board, etc.). Please also provide contact information for a data access committee, ethics committee, or other institutional body to which data requests may be sent.

b) If there are no restrictions, please upload the minimal anonymized data set necessary to replicate your study findings to a stable, public repository and provide us with the relevant URLs, DOIs, or accession numbers. For a list of recommended repositories, please see
<https://journals.plos.org/plosone/s/recommended-repositories>

You also have the option of uploading the data as Supporting Information files, but we would recommend depositing data directly to a data repository if possible. We will update your Data Availability statement on your behalf to reflect the information you provide.

As requested, the image datasets generated and analyzed during this study have been deposited in the BioImage Archive and will be publicly available, starting 06/08/2026, under accession number S-BIAD3547.

7. If the reviewer comments include a recommendation to cite specific previously published works, please review and evaluate these publications to determine whether they are relevant and should be cited. There is no requirement to cite these works unless the editor has indicated otherwise.

N/A

**Reply to the Reviewers**

**Reviewer #1**

The manuscript presents Bo-Net, a deep learning-based pipeline for automatic segmentation and quantification of bone stromal components (osteoblasts, osteoclasts, vasculature, and bone matrix) in fluorescence microscopy images. The study addresses an important bottleneck in analyzing microscopy-based data of bone biology, namely the labor-intensive and subjective nature of manual quantification. The work is valuable to the bone imaging community.

Advantages

1. The study tackles a real and common problem in microscopy: manual image analysis is slow and subjective, and automated tools are needed.
2. The tool detects expected biological effects (aging, drug treatment, tumor changes), which increases confidence in usefulness.
3. Works across both confocal and multiphoton datasets.
4. Potentially valuable for routine use by biology labs without computational expertise.

We thank Reviewer #1 for the positive comments and for recognizing the significance and utility of our work. We appreciate the acknowledgment that Bo-Net addresses the challenge of slow and subjective manual image analysis, detects expected biological effects, performs across both confocal and multiphoton datasets, and has potential value for routine use in biology laboratories without extensive computational expertise.

We addressed the suggested improvements, as follows:

1. Please explain more clearly how the “ground truth” annotations were generated.
   a. Whether more than one person annotated images? If so, does their individual bias affect the model training?

a. We thank the Reviewer for these important questions. The training images were annotated independently by three trained annotators following the same annotation guidelines and quality-control procedures. Using multiple annotators helped reduce the likelihood of individual annotation bias and improved the robustness and generalizability of the training dataset by incorporating variability in human interpretation. In addition, annotations were reviewed for consistency prior to model training to ensure uniform labeling across the dataset.

We added these considerations in the methods, as follows (page 10):

“*Training images were independently annotated by three trained annotators according to standardized annotation guidelines and quality-control procedures. The use of multiple annotators was intended to minimize individual annotation bias and improve the robustness and generalizability of the training dataset by incorporating inter-annotator variability. Prior to model training, all annotations were reviewed for consistency to ensure uniform labeling across the dataset*.”

b. Is there enough training data to account for variations in samples?

b. We believe that the training dataset was sufficiently large and diverse to account for variations across samples and imaging conditions. In total, 21,395 individual images were generated and pre-processed for model development, including 17,104 images used for training and 4,291 images used for validation. The dataset incorporated multiple biological conditions and imaging variations, which helped improve the robustness and generalizability of the model.

We added these considerations in the methods, as follows (page 7)

*“Overall, the dataset incorporated multiple biological conditions and imaging variations, which helped improve the robustness and generalizability of the model”.*

c. Will the user have to generate their own training data since everyone will acquire data differently?

c. We acknowledge that imaging conditions may vary between laboratories due to differences in microscope systems, acquisition settings, staining protocols, and sample preparation. Bo-Net was trained using a large dataset based on acquisition with a confocal and a multiphoton microscope, which improved its robustness to such variability. We anticipate that the pretrained model will generalize well to datasets acquired under similar imaging conditions. Nevertheless, for substantially different imaging setups or staining protocols, additional user-generated annotations and model fine-tuning may further optimize performance.

We added these considerations in the discussion, as follows (page 25)

*“We acknowledge that imaging conditions may vary between laboratories due to differences in microscope systems, acquisition settings, staining protocols, and sample preparation. Bo-Net was trained using a large dataset based on acquisition with a confocal and a multiphoton microscope, which improved its robustness to such variability. We anticipate that the pretrained model will generalize well to datasets acquired under similar imaging conditions. Nevertheless, for substantially different imaging setups or staining protocols, additional user-generated annotations and model fine-tuning may further optimize performance”.*

2. How sensitive is the tool to differences in staining quality or imaging conditions?

We thank the Reviewer for raising this point. In Figure 3B, we compared two different labeling approaches for the same marker (TRAP) using both endogenous fluorescence from a fluorescent reporter mouse and antibody-based staining within the same samples. No significant differences were observed, despite the fluorescent reporter producing a brighter signal with lower background compared to the antibody staining. Similarly, in Figure 6A, no significant differences were detected when comparing endomucin and laminin staining for blood vessel analysis.

In contrast, Figure 3A shows differences between two osteoblast markers, alkaline phosphatase (antibody-based staining) and Sp7 (fluorescent reporter mouse). We believe these differences are primarily due to the distinct biological expression patterns of the two markers. Alkaline phosphatase is membrane-associated, whereas Sp7 is a nuclear transcription factor that is also expressed in osteocytes. These differences in cellular localization and expression likely account for the observed variation in signal and segmentation output.

Overall, these findings suggest that Bo-Net is robust to differences in staining quality and imaging conditions when labeling strategies target similar biological structures, while differences in marker biology may influence segmentation outcomes.

We added these considerations in the discussion, as follows (page 26)

*“Our comparative analyses demonstrate that Bo-Net is robust across different staining strategies and imaging conditions when markers label the same biological structures. Specifically, no significant differences were observed between fluorescent reporter- and antibody-based TRAP labeling, or between endomucin and laminin staining for vascular analysis, despite differences in signal intensity and background levels. In contrast, discrepancies observed between alkaline phosphatase and Sp7 labeling likely reflect intrinsic biological differences between these osteoblast markers, including their distinct cellular localization and expression patterns. Together, these findings indicate that segmentation performance is primarily influenced by marker biology rather than technical variations in staining approach, supporting the adaptability of Bo-Net across diverse experimental settings.”*

3. Microscopy acquisition details are lacking. What objective was used? Parameters - laser powers, pixel dwell time, etc.?

More details have been added to the Materials and Methods, as follows (page 6-7):

*“Samples were acquired using a confocal microscope (Leica SP8) or a custom-made multiphoton microscope (LaVision BioTech).*

*“The multiphoton microscope is equipped with three Ti:Sapphire lasers (Chameleon-XR, Coherent) and two optical parametric oscillators (APE/Coherent), resulting in a tunable excitation range (800-1300 nm). Images were acquired using a 16x water objective (numerical aperture, 0.8, Olympus) and multispectral detection was performed using up to five backward or two forward photomultipliers and up to three excitation wavelengths in two consecutive scans, to separate the following excitation and emission channels: AlexaFluor 488 (920 nm; 525/50 nm), AlexaFluor 594 (1090 nm; 595/40 nm), SHG (1090 nm; 525/50 nm), AlexaFluor 680 (1280 nm; 710/75 nm). Volumes of 360 × 360 μm (1064 × 1064 pixels; frequency 400; line average, 2; laser power 2-20%) with in-between slices z-depth of 8 μm, for a maximum depth of ~80-100 μm were acquired.*

*The Leica SP8 confocal microscope equipped with a Z6 APO zoom system. Images were acquired using a 20x objective at a resolution of 608 x 608 µm (1024 × 1024 pixels), with in-between slices z-depth of 8 μm, for a maximum depth of ~80-100 μm Scanning was performed at a speed of 400 Hz, pinhole set to 1 Airy Unit, with no frame averaging, and identical acquisition settings were maintained across all samples. Laser power was optimized for each fluorophore to achieve sufficient signal while avoiding saturation and photobleaching, and detector gain was adjusted accordingly.”*

**Reviewer #2**

The manuscript by Allegri et al. presents the development of BoNET, a deep learning strategy to segment and analyse fluorescent images of bone (cell) samples from mouse. While BoNET is well described, optimized and tested during the development of the program, the potential and limitations for its use in biological questions remains largely open. Most importantly, a comparison of the results obtained by BoNET versus a classical (manual) analysis for Fig. 4, 5 and 6 is missing. In addition, image acquisition conditions and parameter analysis (at least for Fig. 4-6) is not well described and could (should) be explored a lot further.

We thank the Reviewer for the thoughtful and constructive comments. We appreciate the recognition that BoNET was well described, optimized, and rigorously tested during development. We also agree that further clarification regarding the biological applications and limitations of the framework, additional comparison with manual analyses, and more detailed reporting of imaging acquisition conditions and parameter analysis would strengthen the manuscript. We have addressed these important points in the revised version as detailed below.

1. To evaluate the usefulness of BoNET in analyzing bone cell samples, it is essential to compare the results obtained with it in the biological experiments (in particular Fig. 4 and Fig. 6) to some type of manual analysis: either something used before or a basic strategy by segmenting images (or drawing outlines) in ImageJ and comparing overlap. Only by this can an estimation of the performance and limitations of BoNET be addressed. In case the number of tibiae per group reflects the number of images acquired per condition (is that the case? otherwise please label better) that should be reasonably easy to do.

Also in Fig. 3 when analyzing antibody staining versus reporter construct such a comparison would be very informative.

Is there an option to quantify the segmentation in Fig. 5? If so, would be good to include.

To further evaluate the performance of BoNET, we performed manual segmentation analyses on the datasets shown in Figures 3A,B, 4A,B, and 6A (now Figure 5A). Manual quantification was carried out using image segmentation/outline-based analysis, and the results were compared directly with BoNET outputs. Across multiple independent biological datasets, including different cellular markers, age groups, and treatment conditions, automated quantification showed strong agreement with manual analysis, with Pearson correlation coefficients ranging 0.64 to 0.98 across the analyzed datasets. These results indicate that the model reliably captures biologically relevant differences across diverse experimental settings; these comparative analyses have now been included in Figure S8 and described in the Results section (page 20, 21, 23, 24).

Figure 5 was intended primarily as a proof-of-concept example to demonstrate the ability of BoNET to accurately segment osteoclasts within osteolytic lesions in a representative case. Because this analysis was performed on a single representative image rather than a biological cohort, quantitative comparisons were not included. This Figure has been now moved to the supplementary section (Figure S9) and the description incorporated to the previous paragraph.

2. The information on image acquisition and on parameter analysis is in part insufficient.

a) On image acquisition: Give information on which objective was used, i.e. x/y (and z pixel) resolution, and in particular which z-distance was used when acquiring the planes for the image stacks.

This is an important point raised by the Reviewer. We updated the information relative to microscopy acquisition in the Materials and Methods, as follows (page 6-7):

*“The multiphoton microscope is equipped with three Ti:Sapphire lasers (Chameleon-XR, Coherent) and two optical parametric oscillators (APE/Coherent), resulting in a tunable excitation range (800-1300 nm). Images were acquired using a 16x water objective (numerical aperture, 0.8, Olympus) and multispectral detection was performed using up to five backward or two forward photomultipliers and up to three excitation wavelengths in two consecutive scans, to separate the following excitation and emission channels: AlexaFluor 488 (920 nm; 525/50 nm), AlexaFluor 594 (1090 nm; 595/40 nm), SHG (1090 nm; 525/50 nm), AlexaFluor 680 (1280 nm; 710/75 nm). Volumes of 360 × 360 μm (1064 × 1064 pixels; frequency 400; line average, 2; laser power 2-20%) with in-between slices z-depth of 8 μm, for a maximum depth of ~80-100 μm were acquired.*

*The Leica SP8 confocal microscope equipped with a Z6 APO zoom system. Images were acquired using a 20x objective at a resolution of 608 x 608 µm (1024 × 1024 pixels), with in-between slices z-depth of 8 μm, for a maximum depth of ~80-100 μm Scanning was performed at a speed of 400 Hz, pinhole set to 1 Airy Unit, with no frame averaging, and identical acquisition settings were maintained across all samples. Laser power was optimized for each fluorophore to achieve sufficient signal while avoiding saturation and photobleaching, and detector gain was adjusted accordingly.”*

Along those lines there would be couple of important questions that should be addressed:

Why were 7 z-planes acquired and how much of the bone sample is covered with this?

When anyway using maximum signal intensity projection, would it also work for less z-planes (maybe with different z-distances) or are more z-planes better?

Image stacks were acquired with a z-step of 8 µm over a maximum depth of approximately 80-100 µm. For analysis, we selected the best 7 consecutive z-planes, corresponding to an imaging depth of approximately 50 µm. This approach was chosen to exclude the most superficial planes, which are often fragmented or irregular due to tissue sectioning artifacts, as well as the deepest planes, where signal intensity decreases because of reduced laser penetration and increased light absorption/scattering within the tissue. Selecting these intermediate planes allowed us to obtain images with more consistent morphology and signal quality for segmentation and analysis within the same volume.

In our case, the choice of seven consecutive z-planes represents a compromise between tissue coverage and image quality. Using fewer planes would reduce the amount of structural information captured in the maximum intensity projection and could increase sensitivity to local variations or missing features within individual planes. Conversely, including additional planes would increase the sampled volume but would also incorporate lower-quality superficial or deep planes, potentially introducing artifacts and reducing the overall consistency of the projected image. In our dataset, seven consecutive planes provided sufficient depth coverage while maintaining robust signal quality across samples. We have clarified this rationale in the revised manuscript, as follows:

*“Seven consecutive intermediate z-planes (8 µm spacing; ~50 µm total depth) were selected for analysis to avoid superficial sectioning artifacts and signal loss in deeper regions, providing an optimal balance between tissue coverage and image quality for maximum-intensity projection within the same volume”, page 7.*

In the histograms in Fig. S1 there is a substantial amount of pixels with saturated intensity values. Has this been considered carefully during image acquisition (and adapted accordingly)? And how much does the intensity (signal-to-noise) matter for the performance of BoNET?

If the Reviewer was referring to the small spike on the x-axis, we apologize for the oversight, as this was an unintended graphical artifact that has now been corrected in the revised figure. We would also like to clarify that during image acquisition, laser power and detector gain settings were adjusted to maximize signal detection while minimizing saturation. However, because some biological structures exhibited very strong fluorescence signals, a limited proportion of saturated pixels might have remained present in certain images. BoNET segmentation is not based on absolute fluorescence intensity measurements, but rather on the identification of clear morphological and spatial signal patterns. Therefore, the presence of some saturated pixels was not necessarily detrimental and, in some cases, helped improve contrast between labeled structures and background. Based on our analyses, moderate differences in signal intensity did not substantially affect segmentation performance, although extremely low signal-to-noise ratios or excessive saturation may reduce segmentation accuracy, as expected for most fluorescence image-analysis pipelines.

To further reduce the impact of variability in intensity and signal-to-noise ratio, all images underwent preprocessing, normalization and thresholding prior to training and analysis. Importantly, the training dataset included heterogenous images, in which gamma and thresholding values were randomly selected within a predefined range to preserve adequate image visibility and quality. This approach was intended to avoid transformations that would generate excessively dark or overly bright images, which could compromise their suitability for neural network training and processing.

“*Although a limited number of saturated pixels were present in some images, segmentation was largely unaffected because it relies on morphological and spatial features rather than absolute intensity values. Preprocessing, normalization, and training with heterogeneous image intensities further enhanced robustness to variations in signal intensity and image quality*”, page 8.

b) On parameter analysis:

How was the number of vessels counted, by number of segmented structures? And if so, was there a cut-off, e.g. minimum size (number of pixels)?

The number of vessels was counted by the number of segmented structures. A minimum size threshold of 10 × 10 pixels was applied, corresponding to approximately 5.5 × 5.5 µm. This cutoff was selected to exclude small artifacts and background noise while retaining the smallest capillary-like structures with a visible lumen that could be reliably identified in our imaging conditions.

*“Vessel number was quantified by counting segmented structures after applying a minimum size threshold of 10 × 10 pixels (≈5.5 × 5.5 µm) to exclude artifacts and background noise”,* page 23.

3. Figure legends should be labelled with more details (e.g. which microscope used for Fig. 3 and 4, which labels / secondary antibodies used for images shown, give number of images together with number of samples).

We updated the figure legends for Fig. 3, 4, and 5 as suggested.

Minor points:

Discussion on performance and limitations of BoNET could be more extensive: What other parameters could be made use of, what about 3D analysis / segmentation, is that planned, feasible or is 2D (max. proj.) sufficient? Include results on comparisons mentioned in Major Point 1.

Text errors:

- Fig. 3B: “n.s,” should be “n.s.”

Done.

- Page 18, first sentence is incomplete

Done.

- Page 20, “BoNET successfully segmented …which lead to pathologic bone resorption” has weird causal connection

We modified the sentence, as follows (page 23):
*“To monitor tumor-induced bone resorption, RENCA renal tumor cells were implanted in mouse tibiae and samples were processed for microscopy-based analysis (S9A Fig). These tumor cells induced OC recruitment, which was successfully segmented by BoNET, revealing areas of pathologic bone resorption and disruption of the mineralized bone structure (S9B Fig).”.*

- Fig. 5B: Unit of “Total area” not specified

Done.

- Page 22, Discussion, second paragraph: “Our automated tool… whether” should be “when”

Done.

**Reviewer #3**

The manuscript presents a tool, based on deep-learning pipelines, for automatic segmentation and quantification of bone stroma components in fluorescence images. The results show an automated framework capable of generating segmentation results comparable to manual expert annotation, reducing drastically analysis time. Overall is very well written, with solid controls and the statistical analysis is very complete.

Bo-Net addresses a relevant problem in bone biology and preclinical cancer research, where manual quantification of microscopy images is time-consuming and prone to inter-observer variability. Integrating deep-learning is a strong point of this manuscript and also the different use of multiple biological conditions to test the tool are very impressive.

We thank the Reviewer for the positive and encouraging comments. We appreciate the recognition that Bo-Net addresses an important challenge in bone biology and preclinical cancer research by reducing the time and variability associated with manual image quantification. We are also grateful for the acknowledgment of the robustness of our controls and statistical analyses, as well as the value of integrating deep-learning approaches across multiple biological conditions. We believe these features strengthen the utility and translational potential of the framework for automated fluorescence image analysis.

1. The manuscript reports 5,530 original images that were expanded to 21,395 through preprocessing and augmentation. However, in Materials and methods it's not specify the number of animals that were used for each experiment (it's only mentioned for the osteoclast depletion experiment). Also, whether images from the same stack or animal appear in both training and validation sets nor whether splitting was done at the image level or animal level.

We revised the Materials and Methods section to clarify the origin and organization of the image dataset used for training and validation. Specifically, the dataset was generated from our historical database of multiphoton and confocal microscopy samples collected over several years, including specimens derived from multiple fluorescent reporter mouse models as well as antibody-based stainings. This approach allowed us to maximize dataset diversity while minimizing the need for additional animal use during the training phase.

*“The dataset was generated from a historical collection of multiphoton and confocal microscopy samples acquired over several years, including tissues derived from multiple fluorescent reporter mouse models and antibody-based stainings. This strategy enabled the inclusion of diverse imaging conditions and labeling approaches while minimizing the need for additional animals for model training”,* page 7.

We also clarified that dataset splitting was performed at the sample level; the same stack was not included in both sets. Importantly, all the bone slices acquired are sectioned at 300-µm thick slices using a vibratome. Differently from acquisition of conventional thin slices (5-10 µm), this allows generating unique and biologically different slices, even if cut consecutively (given that the size of the murine cells of interest varies between 20-50 µm for osteoblasts, to 100 µm for osteoclasts, to 50-100 µm diameter for blood vessels), as specified in the Materials and Methods (page 6). Thus, no image, patch, or augmented derivative from the same microscopy stack appeared in both training and validation datasets, page 10:

*“Train-Validation split. The dataset was divided into 80% training and a 20% validation subset at the sample level; the same stack was never included in both sets. As mentioned in the paragraph ”sample staining and acquisition”, bone samples were sectioned into 300-µm-thick vibratome slices, such that even consecutive sections represented distinct tissue volumes and therefore provided biologically unique information. To avoid data leakage and ensure biological independence between training and validation data, the split was performed before patch extraction and data augmentation. Following split, patch extraction and data augmentation were performed independently within each subset. This procedure yielded a final dataset of 21,395 images, including 17,104 images for training and 4,291 images for validation.”*

2. The performance evaluation was done with only 5 images per object class, this in my view is a rather small set. It could be an improvement to expand the evaluation to multiple independent biological experiments and include the confidence intervals for evaluation metrics.

We apologize for the mistake. The evaluation was done on 8-10 images, as now updated in the figure legend. We further expanded the validation by performing manual analysis of data reported in Fig. 3, 4 and 5, and identified a Pearson correlation between BoNet and manual analysis spanning 0.64-0.98, as shown in supplementary Fig. S8. These results indicate that the model reliably captures biologically relevant differences across diverse experimental settings; these comparative analyses have now been described in the Results section (page 20, 21, 23, 24).

3. The authors say that there is no automatic tool currently available for this specific task, but the study would benefit from a clearer comparison with existing bioimage segmentation tools, maybe Cellpose or StarDist. It could be interesting to compare a small dataset for one or two markers using an available segmentation tool and check the improvement by using Bo-Net.

We were already familiar with StarDist, a deep learning–based detection and segmentation method that we have frequently used in our work for nuclei segmentation. However, this approach is not well suited to our images, which predominantly contain flat linear structures or irregularly shaped objects rather than round cells or nuclei.

We were not previously familiar with Cellpose, but we tested it and obtained the results shown in Fig. R1.

Despite multiple optimization attempts and adjustment of input parameters, segmentation quality remained suboptimal. In the Sp7 image, where the marker is expressed by both osteoblasts and osteocytes, Cellpose preferentially segmented osteocytes, which were not the primary cells of interest, while failing to accurately identify osteoblasts lining the bone surface. Similarly, for blood vessel analysis, repeated parameter optimization did not substantially improve vessel recognition, which remained inadequate. Similar to StarDist, Cellpose is primarily designed for round or compact cellular morphologies, which likely limits its applicability to our image type.

**Fig. R1. Segmentation with Cellpose.** Original pictures and the result of Cellpose segmentation is shown for osteoblasts (OB) and blood vessels. Bar, 200 µm.

4. The manuscript states that data are available upon request and the model will be released on GitHub after publication. I would encourage the authors to share sample annotated datasets in a public repository after publication.

The image datasets generated and analyzed during this study (including the annotated database) have been deposited in the BioImage Archive and will be publicly available under accession number S-BIAD3547. Bo-Net source code/software is also already available on GitHub at following link: <https://github.com/scasarin87/Bo-Net>

5. Methods describing preprocessing and weighting strategies are very detailed and technical. A brief summary diagram of the computational workflow could help readers follow the pipeline more easily.

We have added a schematic overview of the Bo-Net computational workflow as suggested in the Materials and Methods section (New Fig. S1).

Minor comment:

In the “Segmentation and quantification of the impact of intrinsic and extrinsic modifications of OBs and OCs by Bo-Net” section it appears that the first sentence is incomplete (at different what? I assume it's time points).

Modified.
