# Supplementary material for: Bo-Net: Deep learning-based model for automatic bone stromal cell segmentation of fluorescence microscopy images
Source: PLoS One. 2026 Aug 4;21(8):e0353796. doi: 10.1371/journal.pone.0353796 (PMC13436720; doi:10.1371/journal.pone.0353796)
Supplement: Supplementary file 2 [file pone.0353796.s012.docx]

**Response to reviewers**

We thank the reviewers for their careful reading of the revised manuscript and helpful comments.

**1. Please standardize the use of Bo-Net versus BoNet throughout the manuscript and choose one form.**

Standardized throughout the manuscript as “Bo-Net”.

**2. In Equation 5, there is a typo: “Acuracy”. Please correct this and carefully check all equations and equation labels.**

Corrected. We also double-checked all equations and equation labels.

**3. Based on reply to reviewer 3, the authors mention they used 8-10 images instead of 5. But Table 7 and other figs still mention 5.**

We corrected the caption of Table 7. After reviewing all figure captions, we did not identify any additional image-number mismatches.

**4. Again based on reply to reviewer 3, the authors say they tested StarDist and Cellpose and found these programs were not suitable for their analysis. This is important information that needs to be in the manuscript to show the utility of their tool.**

We added this information to the Discussion section (page 22). We now explain that StarDist and Cellpose were not suitable for our dataset because the structures of interest are predominantly linear, irregular, or tissue-associated rather than round or compact cellular objects. We also report that, despite parameter optimization, Cellpose preferentially segmented osteocytes in Sp7 images and did not adequately recognize blood vessels. Added part reads

*“Currently available general-purpose image segmentation algorithm do not directly perform differential segmentation and quantification of multiple bone stromal cells and bone within the same fluorescence microscopy image. In line with this, we evaluated the applicability of StarDist and Cellpose, two widely used tools, to representative images from our dataset. StarDist was not well suited to our images, which predominantly contain flat, linear structures or irregularly shaped objects rather than round cells or nuclei. We also tested Cellpose using representative images of OB and BV, and even after several optimization attempts involving the adjustment of input parameters, the segmentation quality remained suboptimal. In Sp7 images, where the marker is expressed by both OBs and osteocytes, Cellpose preferentially segmented osteocytes rather than OBs lining the bone surface, which were the cells of interest. Similarly, for BV analysis, repeated parameter optimization did not substantially improve vessel recognition. These observations suggest that, despite their broad utility, currently available generalist tools have limited applicability regarding the specific morphologies detected in this study, further supporting the utility of Bo-Net”.*
