# Supplementary material for: Bo-Net: Deep learning-based model for automatic bone stromal cell segmentation of fluorescence microscopy images
Source: PLoS One. 2026 Aug 4;21(8):e0353796. doi: 10.1371/journal.pone.0353796 (PMC13436720; doi:10.1371/journal.pone.0353796)
Supplement: Peer review history

# Peer review: Decision Letter 0

**DOI:** 10.1371/journal.pone.0353796.r001

**Authors:** Tomasz Kaminski

11 Mar 2026

PONE-D-26-03682

Bo-Net: Deep Learning-based Model for Automatic Bone Stromal Cell Segmentation of Fluorescence Microscopy Images

PLOS One

Dear Dr. Casarin,

Thank you for submitting your manuscript to PLOS ONE. After careful consideration, we feel that it has merit but does not fully meet PLOS ONE’s publication criteria as it currently stands. Therefore, we invite you to submit a revised version of the manuscript that addresses the points raised during the review process.

Please submit your revised manuscript by Apr 25 2026 11:59PM. If you will need more time than this to complete your revisions, please reply to this message or contact the journal office at plosone@plos.org. When you're ready to submit your revision, log on to https://www.editorialmanager.com/pone/ and select the 'Submissions Needing Revision' folder to locate your manuscript file.

Please include the following items when submitting your revised manuscript:

- A letter that responds to each point raised by the academic editor and reviewer(s). You should upload this letter as a separate file labeled 'Response to Reviewers'.
- A marked-up copy of your manuscript that highlights changes made to the original version. You should upload this as a separate file labeled 'Revised Manuscript with Track Changes'.
- An unmarked version of your revised paper without tracked changes. You should upload this as a separate file labeled 'Manuscript'.

If you would like to make changes to your financial disclosure, please include your updated statement in your cover letter. Guidelines for resubmitting your figure files are available below the reviewer comments at the end of this letter.

If applicable, we recommend that you deposit your laboratory protocols in protocols.io to enhance the reproducibility of your results. Protocols.io assigns your protocol its own identifier (DOI) so that it can be cited independently in the future. For instructions see: https://journals.plos.org/plosone/s/submission-guidelines#loc-laboratory-protocols. Additionally, PLOS ONE offers an option for publishing peer-reviewed Lab Protocol articles, which describe protocols hosted on protocols.io. Read more information on sharing protocols at https://plos.org/protocols?utm_medium=editorial-email&utm_source=authorletters&utm_campaign=protocols.

We look forward to receiving your revised manuscript.

Kind regards,

Tomasz W. Kaminski

Academic Editor

PLOS One

Journal Requirements:

When submitting your revision, we need you to address these additional requirements.

1. Please ensure that your manuscript meets PLOS ONE's style requirements, including those for file naming. The PLOS ONE style templates can be found at

https://journals.plos.org/plosone/s/file?id=wjVg/PLOSOne_formatting_sample_main_body.pdf and

https://journals.plos.org/plosone/s/file?id=ba62/PLOSOne_formatting_sample_title_authors_affiliations.pdf

2. Please note that PLOS One has specific guidelines on code sharing for submissions in which author-generated code underpins the findings in the manuscript. In these cases, all author-generated code must be made available without restrictions upon publication of the work. Please review our guidelines at https://journals.plos.org/plosone/s/materials-and-software-sharing#loc-sharing-code and ensure that your code is shared in a way that follows best practice and facilitates reproducibility and reuse

3.Thank you for stating the following in the Acknowledgments Section of your manuscript:

" Animal studies were approved by the Institutional Animal Care and Use Committee of the University of Texas, MD Anderson Cancer Center, which is accredited by the Association for Assessment and Accreditation of Laboratory Animal Care. We thank Dr. Masaru Ishii for sharing TRAP-tD-tomato mice (Osaka University, Osaka, Japan). We thank Dr. Ralph Adams (Max Planck Institute for Molecular Biomedicine, Münster, Germany) for sharing mCherry-Sp7 mice. This work was supported by the Cancer Prevention and Research Institute of Texas (RP230160), the National Institutes of Health (R21 CA267312-01A1), the David H. Koch Center for Applied Research of Genitourinary Cancers, The University of Texas MD Anderson Cancer Center, and the John F. Jr. and Carolyn Bookout Presidential Distinguished Chair fund. The Genitourinary Cancers Program of the CCSG shared resources at The University of Texas MD Anderson Cancer Center was supported by the National Cancer Institute at the National Institutes of Health (P30 CA016672). The funders had no role in study design, data collection and analysis, decision to publish, or preparation of the manuscript."

We note that you have provided additional information within the Acknowledgements Section that is not currently declared in your Funding Statement. Please note that funding information should not appear in the Acknowledgments section or other areas of your manuscript. We will only publish funding information present in the Funding Statement section of the online submission form.

Please remove any funding-related text from the manuscript and let us know how you would like to update your Funding Statement. Currently, your Funding Statement reads as follows:

"This work was supported by the Cancer Prevention and Research Institute of Texas (RP230160), the National Institutes of Health (R21 CA267312-01A1), the David H. Koch Center for Applied Research of Genitourinary Cancers, The University of Texas MD Anderson Cancer Center, and the John F. Jr. and Carolyn Bookout Presidential Distinguished Chair fund. The Genitourinary Cancers Program of the CCSG shared resources at The University of Texas MD Anderson Cancer Center was supported by the National Cancer Institute at the National Institutes of Health (P30 CA016672)"

Please include your amended statements within your cover letter; we will change the online submission form on your behalf.

4.Thank you for stating the following financial disclosure:

"This work was supported by the Cancer Prevention and Research Institute of Texas (RP230160), the National Institutes of Health (R21 CA267312-01A1), the David H. Koch Center for Applied Research of Genitourinary Cancers, The University of Texas MD Anderson Cancer Center, and the John F. Jr. and Carolyn Bookout Presidential Distinguished Chair fund. The Genitourinary Cancers Program of the CCSG shared resources at The University of Texas MD Anderson Cancer Center was supported by the National Cancer Institute at the National Institutes of Health (P30 CA016672)"

Please state what role the funders took in the study.  If the funders had no role, please state: "The funders had no role in study design, data collection and analysis, decision to publish, or preparation of the manuscript."

If this statement is not correct you must amend it as needed.

Please include this amended Role of Funder statement in your cover letter; we will change the online submission form on your behalf.

5. Your ethics statement should only appear in the Methods section of your manuscript. If your ethics statement is written in any section besides the Methods, please delete it from any other section.

6. We note that you have indicated that there are restrictions to data sharing for this study. PLOS only allows data to be available upon request if there are legal or ethical restrictions on sharing data publicly. For more information on unacceptable data access restrictions, please see http://journals.plos.org/plosone/s/data-availability#loc-unacceptable-data-access-restrictions.

Before we proceed with your manuscript, please address the following prompts:

a) If there are ethical or legal restrictions on sharing a de-identified data set, please explain them in detail (e.g., data contain potentially identifying or sensitive patient information, data are owned by a third-party organization, etc.) and who has imposed them (e.g., a Research Ethics Committee or Institutional Review Board, etc.). Please also provide contact information for a data access committee, ethics committee, or other institutional body to which data requests may be sent.

b) If there are no restrictions, please upload the minimal anonymized data set necessary to replicate your study findings to a stable, public repository and provide us with the relevant URLs, DOIs, or accession numbers. For a list of recommended repositories, please see

https://journals.plos.org/plosone/s/recommended-repositories. You also have the option of uploading the data as Supporting Information files, but we would recommend depositing data directly to a data repository if possible.

We will update your Data Availability statement on your behalf to reflect the information you provide.

7. If the reviewer comments include a recommendation to cite specific previously published works, please review and evaluate these publications to determine whether they are relevant and should be cited. There is no requirement to cite these works unless the editor has indicated otherwise.

Additional Editor Comments:

Dear Authors,

Thank you for submitting your manuscript on the Bo-Net segmentation framework. The reviewers found the study interesting and potentially valuable for the bone imaging community, particularly because it addresses the important challenge of automated analysis of fluorescence microscopy data. However, several important points need to be clarified and strengthened, including validation against manual quantification, clearer description of the training/annotation strategy and dataset splitting, and more detailed reporting of imaging acquisition parameters; therefore, we invite you to submit a revised version addressing the reviewers’ comments.

Best regards,

Tomasz W Kaminski

[Note: HTML markup is below. Please do not edit.]

Reviewers' comments:

Reviewer's Responses to Questions

**Comments to the Author**

1. Is the manuscript technically sound, and do the data support the conclusions?

The manuscript must describe a technically sound piece of scientific research with data that supports the conclusions. Experiments must have been conducted rigorously, with appropriate controls, replication, and sample sizes. The conclusions must be drawn appropriately based on the data presented.

Reviewer #1: Yes

Reviewer #2: Partly

Reviewer #3: Yes

**********

2. Has the statistical analysis been performed appropriately and rigorously?

Reviewer #1: Yes

Reviewer #2: Yes

Reviewer #3: Yes

**********

3. Have the authors made all data underlying the findings in their manuscript fully available?

The PLOS Data policy requires authors to make all data underlying the findings described in their manuscript fully available without restriction, with rare exception (please refer to the Data Availability Statement in the manuscript PDF file). The data should be provided as part of the manuscript or its supporting information, or deposited to a public repository. For example, in addition to summary statistics, the data points behind means, medians and variance measures should be available. If there are restrictions on publicly sharing data—e.g. participant privacy or use of data from a third party—those must be specified.

Reviewer #1: Yes

Reviewer #2: Yes

Reviewer #3: Yes

**********

4. Is the manuscript presented in an intelligible fashion and written in standard English?

PLOS ONE does not copyedit accepted manuscripts, so the language in submitted articles must be clear, correct, and unambiguous. Any typographical or grammatical errors should be corrected at revision, so please note any specific errors here.

Reviewer #1: Yes

Reviewer #2: Yes

Reviewer #3: Yes

**********

5. Review Comments to the Author

Please use the space provided to explain your answers to the questions above. You may also include additional comments for the author, including concerns about dual publication, research ethics, or publication ethics. (Please upload your review as an attachment if it exceeds 20,000 characters)

Reviewer #1: The manuscript presents Bo-Net, a deep learning-based pipeline for automatic segmentation and quantification of bone stromal components (osteoblasts, osteoclasts, vasculature, and bone matrix) in fluorescence microscopy images. The study addresses an important bottleneck in analyzing microscopy-based data of bone biology, namely the labor-intensive and subjective nature of manual quantification. The work is valuable to the bone imaging community.

Advantages

1. The study tackles a real and common problem in microscopy: manual image analysis is slow and subjective, and automated tools are needed.

2. The tool detects expected biological effects (aging, drug treatment, tumor changes), which increases confidence in usefulness.

3. Works across both confocal and multiphoton datasets.

4. Potentially valuable for routine use by biology labs without computational expertise.

Suggested Improvements

1. Please explain more clearly how the “ground truth” annotations were generated.

a. Whether more than one person annotated images? If so, does their individual bias affect the model training?

b. Is there enough training data to account for variations in samples?

c. Will the user have to generate their own training data since everyone will acquire data differently?

2. How sensitive is the tool to differences in staining quality or imaging conditions?

3. Microscopy acquisition details are lacking. What objective was used? Parameters - laser powers, pixel dwell time, etc.?

Reviewer #2: Bo-Net Manuscript – Comments

The manuscript by Allegri et al. presents the development of BoNET, a deep learning strategy to segment and analyse fluorescent images of bone (cell) samples from mouse.

While BoNET is well described, optimized and tested during the development of the program, the potential and limitations for its use in biological questions remains largely open. Most importantly, a comparison of the results obtained by BoNET versus a classical (manual) analysis for Fig. 4, 5 and 6 is missing. In addition, image acquisition conditions and parameter analysis (at least for Fig. 4-6) is not well described and could (should) be explored a lot further.

Major Points:

1. To evaluate the usefulness of BoNET in analyzing bone cell samples, it is essential to compare the results obtained with it in the biological experiments (in particular Fig. 4 and Fig. 6!) to some type of manual analysis: either something used before or a basic strategy by segmenting images (or drawing outlines) in ImageJ and comparing overlap. Only by this, an estimation of the performance and limitations of BoNET can be addressed. In case the number of tibiae per group reflects the number of images acquired per condition (is that the case? otherwise please label better) that should be reasonably easy to do.

Also in Fig. 3 when analyzing antibody staining versus reporter construct such a comparison would be very informative!

Is there an option to quantify the segmentation in Fig.5? If so, would be good to include!

2. The information on image acquisition and on parameter analysis is in part insufficient

a) On image acquisition: Give information on which objective was used, i.e. x/y (and z pixel) resolution, and in particular which z-distance was used when acquiring the planes for the image stacks.

Along those lines there would be couple of important questions that should be addressed:

Why were 7 z-planes acquired and how much of the bone sample is covered with this?

When anyway using maximum signal intensity projection, would it also work for less z-planes (maybe with different z-distances) or are more z-planes better?

In the histograms in Fig. S1 there is a substantial amount of pixels with saturated intensity values => Has this been considered carefully during image acquisition (and adapted accordingly)? And how much does the intensity (signal-to-noise) matter for the performance of BoNET?

b) On parameter analysis:

How was the number of vessels counted, by number of segmented structures? And if so, was there a cut-off, e.g. minimum size (number of pixels)?

3. Figure legends should be labelled with more details (e.g. which microscope used for Fig. 3 and 4, which labels / secondary antibodies used for images shown, give number of images together with number of samples)

Minor points:

Discussion on performance and limitations of BoNET could be more extensive: What other parameters could be made use of, what about 3D analysis / segmentation, is that planned, feasible or is 2D (max. proj.) sufficient? -> Include results on comparisons mentioned in Major Point 1.

Text errors:

- Fig. 3B: “n.s,” should be “n.s.”

- Page 18, first sentence is incomplete

- Page 20, “BoNET successfully segmented …which lead to pathologic bone resorption” has weird causal connection

- Fig. 5B: Unit of “Total area” not specified

- Page 22, Discussion, second paragraph: “Our automated tool… whether” -> should be “when”

Reviewer #3: The manuscript presents a tool, based on deep-learning pipelines, for automatic segmentation and queantification of bone stroma components in fluorescence images. The results show an automated framework capable of generating segmentation results comparable to manual expert annotation, reducing drasticaly analysis time. Overall is very well written, with solid controls and the statistical analysis is very complete.

Bo-Net addresses a relevant problem in bone biology and preclinical cancer research, where manual quantification of microscopy images is time-consuming and prone to inter-observer variability. Integrating deep-learining is a strong point of this manuscript and also the different use of multiple biological conditions to test the tool are very impresive.

Comments:

1- The manuscript reports 5,530 original images that were expanded to 21,395 through preprocessing and augmentation. However, in Materials and methods it's not specify the number of animals that were used for each experiment (it's only mentioned for the steoclast depletion experiment). Also, whether images from the same stack or animal appear in both training and validation sets nor whether splitting was done at the image level or animal level.

2- The performance evaluation was done with only 5 images per object class, this in my view is a rather small set. It could be an improvement to expand the evaluation to multiple independent biological experiments and include the confidence intervals for evaluation metrics.

3- The authors say that there is no automatic tool currently available for this specific task, but the study would benefit from a clearer comparison with existing bioimage segmentation tools, maybe Cellpose or StarDist. It could be interesting to compare a small dataset for one or two markers using an available segmentation tool and check the improvement by using Bo-Net.

4-The manuscript states that data are available upon request and the model will be released on GitHub after publication. I would encourage the authors to share sample annotated datasets in a public repository after publication.

5- Methods describing preprocessing and weighting strategies are very detailed and technical. A brief summary diagram of the computational workflow could help readers follow the pipeline more easily.

Minor comment:

In the Segmentation and quantification of the impact of intrinsic and extrinsic modifications of OBs and OCs by Bo-Net section it appears that the first sentence is incomplete (at different what? I assume it's time points).

**********

6. PLOS authors have the option to publish the peer review history of their article (what does this mean?). If published, this will include your full peer review and any attached files.

If you choose “no”, your identity will remain anonymous but your review may still be made public.

**Do you want your identity to be public for this peer review?**  For information about this choice, including consent withdrawal, please see our Privacy Policy.

Reviewer #1: No

Reviewer #2: **Yes:** Christian Schuberth

Reviewer #3: No

**********

[NOTE: If reviewer comments were submitted as an attachment file, they will be attached to this email and accessible via the submission site. Please log into your account, locate the manuscript record, and check for the action link "View Attachments". If this link does not appear, there are no attachment files.]

To ensure your figures meet our technical requirements, please review our figure guidelines: https://journals.plos.org/plosone/s/figures

You may also use PLOS’s free figure tool, NAAS, to help you prepare publication quality figures: https://journals.plos.org/plosone/s/figures#loc-tools-for-figure-preparation.

NAAS will assess whether your figures meet our technical requirements by comparing each figure against our figure specifications.

# Author comment: Author response to Decision Letter 1

**DOI:** 10.1371/journal.pone.0353796.r002

13 Jun 2026

We included point-by-point rebuttal in the re-submission.

Attachment Submitted filename: Response to Reviewers.docx

# Peer review: Decision Letter 1

**DOI:** 10.1371/journal.pone.0353796.r003

**Authors:** Tomasz Kaminski

22 Jun 2026

PONE-D-26-03682R1

Bo-Net: Deep Learning-based Model for Automatic Bone Stromal Cell Segmentation of Fluorescence Microscopy Images

PLOS One

Dear Dr. Casarin,

Thank you for submitting your manuscript to PLOS ONE. After careful consideration, we feel that it has merit but does not fully meet PLOS ONE’s publication criteria as it currently stands. Therefore, we invite you to submit a revised version of the manuscript that addresses the points raised during the review process.

Please submit your revised manuscript by Aug 06 2026 11:59PM. If you will need more time than this to complete your revisions, please reply to this message or contact the journal office at plosone@plos.org. When you're ready to submit your revision, log on to https://www.editorialmanager.com/pone/ and select the 'Submissions Needing Revision' folder to locate your manuscript file.

Please include the following items when submitting your revised manuscript:

- A letter that responds to each point raised by the academic editor and reviewer(s). You should upload this letter as a separate file labeled 'Response to Reviewers'.
- A marked-up copy of your manuscript that highlights changes made to the original version. You should upload this as a separate file labeled 'Revised Manuscript with Track Changes'.
- An unmarked version of your revised paper without tracked changes. You should upload this as a separate file labeled 'Manuscript'.

If you would like to make changes to your financial disclosure, please include your updated statement in your cover letter. Guidelines for resubmitting your figure files are available below the reviewer comments at the end of this letter.

If applicable, we recommend that you deposit your laboratory protocols in protocols.io to enhance the reproducibility of your results. Protocols.io assigns your protocol its own identifier (DOI) so that it can be cited independently in the future. For instructions see: https://journals.plos.org/plosone/s/submission-guidelines#loc-laboratory-protocols. Additionally, PLOS ONE offers an option for publishing peer-reviewed Lab Protocol articles, which describe protocols hosted on protocols.io. Read more information on sharing protocols at https://plos.org/protocols?utm_medium=editorial-email&utm_source=authorletters&utm_campaign=protocols.

As the corresponding author, your ORCID iD is verified in the submission system and will appear in the published article. PLOS supports the use of ORCID, and we encourage all coauthors to register for an ORCID iD and use it as well. Please encourage your coauthors to verify their ORCID iD within the submission system before final acceptance, as unverified ORCID iDs will not appear in the published article. *Only*  the individual author can complete the verification step; PLOS staff *cannot*  verify ORCID iDs on behalf of authors.

We look forward to receiving your revised manuscript.

Kind regards,

Tomasz W. Kaminski

Academic Editor

PLOS One

Journal Requirements:

If the reviewer comments include a recommendation to cite specific previously published works, please review and evaluate these publications to determine whether they are relevant and should be cited. There is no requirement to cite these works unless the editor has indicated otherwise.

Please review your reference list to ensure that it is complete and correct. If you have cited papers that have been retracted, please include the rationale for doing so in the manuscript text, or remove these references and replace them with relevant current references. Any changes to the reference list should be mentioned in the rebuttal letter that accompanies your revised manuscript. If you need to cite a retracted article, indicate the article’s retracted status in the References list and also include a citation and full reference for the retraction notice.

Additional Editor Comments:

Dear Authors,

Thank you for submitting the revised version of your manuscript. I am pleased to note that two reviewers recommended acceptance, and one reviewer recommended only minor revision. Overall, the reviewers agree that the manuscript has been substantially improved and that the main concerns have been adequately addressed.

Before the manuscript can be accepted, please address the following minor points:

1. Please standardize the use of Bo-Net versus BoNet throughout the manuscript and choose one form.

2. In Equation 5, there is a typo: “Acuracy”. Please correct this and carefully check all equations and equation labels.

3. In the response to Reviewer 3, the authors mention that 8-10 images were used instead of 5. However, Table 7 and some figures still refer to 5 images. Please make this consistent throughout the manuscript.

4. In the response to Reviewer 3, the authors state that StarDist and Cellpose were tested but were not suitable for this analysis. This is important information and should be included in the manuscript, as it helps demonstrate the utility and added value of the proposed tool.

Please submit a revised version addressing these minor corrections. Provided these points are satisfactorily resolved, the manuscript should be suitable for acceptance.

Best regards,

Tomasz W Kaminski

[Note: HTML markup is below. Please do not edit.]

Reviewers' comments:

Reviewer's Responses to Questions

**Comments to the Author**

1. If the authors have adequately addressed your comments raised in a previous round of review and you feel that this manuscript is now acceptable for publication, you may indicate that here to bypass the “Comments to the Author” section, enter your conflict of interest statement in the “Confidential to Editor” section, and submit your "Accept" recommendation.

Reviewer #1: All comments have been addressed

Reviewer #2: All comments have been addressed

Reviewer #3: All comments have been addressed

**********

2. Is the manuscript technically sound, and do the data support the conclusions?

The manuscript must describe a technically sound piece of scientific research with data that supports the conclusions. Experiments must have been conducted rigorously, with appropriate controls, replication, and sample sizes. The conclusions must be drawn appropriately based on the data presented.

Reviewer #1: Yes

Reviewer #2: Yes

Reviewer #3: Yes

**********

3. Has the statistical analysis been performed appropriately and rigorously?

Reviewer #1: I Don't Know

Reviewer #2: Yes

Reviewer #3: Yes

**********

4. Have the authors made all data underlying the findings in their manuscript fully available?

The PLOS Data policy requires authors to make all data underlying the findings described in their manuscript fully available without restriction, with rare exception (please refer to the Data Availability Statement in the manuscript PDF file). The data should be provided as part of the manuscript or its supporting information, or deposited to a public repository. For example, in addition to summary statistics, the data points behind means, medians and variance measures should be available. If there are restrictions on publicly sharing data—e.g. participant privacy or use of data from a third party—those must be specified.

Reviewer #1: Yes

Reviewer #2: Yes

Reviewer #3: Yes

**********

5. Is the manuscript presented in an intelligible fashion and written in standard English?

PLOS ONE does not copyedit accepted manuscripts, so the language in submitted articles must be clear, correct, and unambiguous. Any typographical or grammatical errors should be corrected at revision, so please note any specific errors here.

Reviewer #1: Yes

Reviewer #2: Yes

Reviewer #3: Yes

**********

6. Review Comments to the Author

Please use the space provided to explain your answers to the questions above. You may also include additional comments for the author, including concerns about dual publication, research ethics, or publication ethics. (Please upload your review as an attachment if it exceeds 20,000 characters)

Reviewer #1: The authors have addressed my comments. The manuscript has been improved by the changes made.

A few minor corrections:

1. Inconsistent use of Bo-Net vs BoNet. Choose one.

2. Eq 5 typo - "Acuracy". Please check and confirm all equations and their labels are correct.

3. Based on reply to reviewer 3, the authors mention they used 8-10 images instead of 5. But Table 7 and other figs still mention 5.

4. Again based on reply to reviewer 3, the authors say they tested StarDist and Cellpose and found these programs were not suitable for their analysis. This is important information that needs to be in the manuscript to show the utility of their tool.

Reviewer #2: The authors did a good job in addressing the points raised during the revision. From my point of view, the manuscript can now be accepted for publication.

Reviewer #3: (No Response)

**********

7. PLOS authors have the option to publish the peer review history of their article (what does this mean?). If published, this will include your full peer review and any attached files.

If you choose “no”, your identity will remain anonymous but your review may still be made public.

**Do you want your identity to be public for this peer review?**  For information about this choice, including consent withdrawal, please see our Privacy Policy.

Reviewer #1: No

Reviewer #2: **Yes:** Dr. Christian Schuberth

Reviewer #3: **Yes:** Victoria Lucia Alonso

**********

[NOTE: If reviewer comments were submitted as an attachment file, they will be attached to this email and accessible via the submission site. Please log into your account, locate the manuscript record, and check for the action link "View Attachments". If this link does not appear, there are no attachment files.]

To ensure your figures meet our technical requirements, please review our figure guidelines: https://journals.plos.org/plosone/s/figures

You may also use PLOS’s free figure tool, NAAS, to help you prepare publication quality figures: https://journals.plos.org/plosone/s/figures#loc-tools-for-figure-preparation.

NAAS will assess whether your figures meet our technical requirements by comparing each figure against our figure specifications.

# Author comment: Author response to Decision Letter 2

**DOI:** 10.1371/journal.pone.0353796.r004

25 Jun 2026

Please see uploaded response to reviewers file

Attachment Submitted filename: Response to reviewers _ r2.docx

# Editor report: Decision Letter 2

**DOI:** 10.1371/journal.pone.0353796.r005

**Authors:** Tomasz Kaminski

29 Jun 2026

Bo-Net: Deep Learning-based Model for Automatic Bone Stromal Cell Segmentation of Fluorescence Microscopy Images

PONE-D-26-03682R2

Dear Dr. Casarin,

We’re pleased to inform you that your manuscript has been judged scientifically suitable for publication and will be formally accepted for publication once it meets all outstanding technical requirements.

Within one week, you’ll receive an e-mail detailing the required amendments. When these have been addressed, you’ll receive a formal acceptance letter and your manuscript will be scheduled for publication.

An invoice will be generated when your article is formally accepted. Please note, if your institution has a publishing partnership with PLOS and your article meets the relevant criteria, all or part of your publication costs will be covered. Please make sure your user information is up-to-date by logging into Editorial Manager at Editorial Manager® and clicking the ‘Update My Information' link at the top of the page. For questions related to billing, please contact billing support.

If your institution or institutions have a press office, please notify them about your upcoming paper to help maximize its impact. If they’ll be preparing press materials, please inform our press team as soon as possible -- no later than 48 hours after receiving the formal acceptance. Your manuscript will remain under strict press embargo until 2 pm Eastern Time on the date of publication. For more information, please contact onepress@plos.org.

Kind regards,

Tomasz W. Kaminski

Academic Editor

PLOS One

# Editor report: Acceptance letter

**DOI:** 10.1371/journal.pone.0353796.r006

**Authors:** Tomasz Kaminski

PONE-D-26-03682R2

PLOS One

Dear Dr. Casarin,

I'm pleased to inform you that your manuscript has been deemed suitable for publication in PLOS One. Congratulations! Your manuscript is now being handed over to our production team.

At this stage, our production department will prepare your paper for publication. This includes ensuring the following:

* All references, tables, and figures are properly cited

* All relevant supporting information is included in the manuscript submission,

* There are no issues that prevent the paper from being properly typeset

You will receive further instructions from the production team, including instructions on how to review your proof when it is ready. Please keep in mind that we are working through a large volume of accepted articles, so please give us a few days to review your paper and let you know the next and final steps.

Lastly, if your institution or institutions have a press office, please let them know about your upcoming paper now to help maximize its impact. If they'll be preparing press materials, please inform our press team within the next 48 hours. Your manuscript will remain under strict press embargo until 2 pm Eastern Time on the date of publication. For more information, please contact onepress@plos.org.

You will receive an invoice from PLOS for your publication fee after your manuscript has reached the completed accept phase. If you receive an email requesting payment before acceptance or for any other service, this may be a phishing scheme. Learn how to identify phishing emails and protect your accounts at https://explore.plos.org/phishing.

If we can help with anything else, please email us at customercare@plos.org.

Thank you for submitting your work to PLOS ONE and supporting open access.

Kind regards,

PLOS ONE Editorial Office Staff

on behalf of

Dr. Tomasz W. Kaminski

Academic Editor

PLOS One
